# Molecular symmetry breaking in the Frizzled-dependent planar polarity pathway

**DOI:** 10.1016/j.cub.2023.10.071

**Published:** 2023-11-22

**Authors:** Helen Strutt, Samantha Warrington, Amritha Chemmenchery Kokkam Madathil, Tobias Langenhan, David Strutt

**Affiliations:** 1 School of Biosciences, University of Sheffield, Firth Court, Sheffield S10 2TN, UK; 2 Rudolf Schönheimer Institute of Biochemistry, Division of General Biochemistry, Medical Faculty, Leipzig University, 04103 Leipzig, Germany

## Abstract

The core planar polarity pathway consists of six proteins that form asymmetric intercellular complexes that segregate to opposite cell ends in developing tissues and specify polarized cell structures or behaviors. Within these complexes, the atypical cadherin Flamingo localizes on both sides of intercellular junctions, where it interacts homophilically in *trans* via its cadherin repeats, whereas the transmembrane proteins Frizzled and Strabismus localize to the opposite sides of apposing junctions. However, the molecular mechanisms underlying the formation of such asymmetric complexes are poorly understood. Using a novel tissue culture system, we determine the minimum requirements for asymmetric complex assembly in the absence of confounding feedback mechanisms. We show that complexes are intrinsically asymmetric and that an interaction of Frizzled and Flamingo in one cell with Flamingo in the neighboring cell is the key symmetry-breaking step. In contrast, Strabismus is unable to promote homophilic Flamingo *trans* binding and is only recruited into complexes once Frizzled has entered on the opposite side. This interaction with Strabismus requires intact intracellular loops of the seven-pass transmembrane domain of Flamingo. Once recruited, Strabismus stabilizes the intercellular complexes together with the three cytoplasmic core proteins. We propose a model whereby Flamingo exists in a closed conformation and binding of Frizzled in one cell results in a conformational change that allows its cadherin repeats to interact with a Flamingo molecule in the neighboring cell. Flamingo in the adjacent cell then undergoes a further change in the seven-pass transmembrane region that promotes the recruitment of Strabismus.

## Graphical abstract

**Figure:**
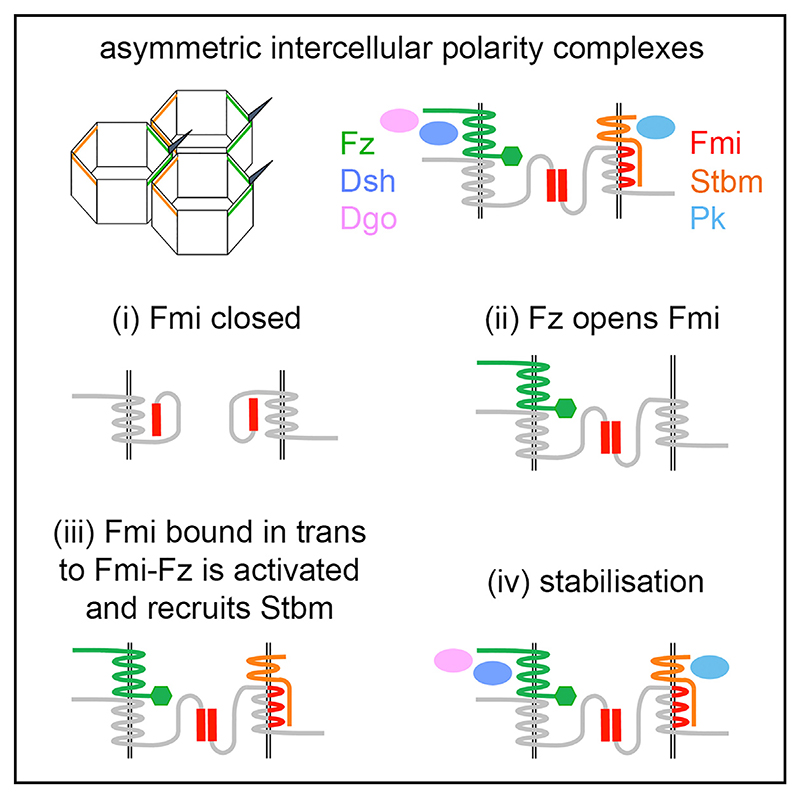
Graphical abstract

## Introduction

Planar polarity describes the phenomenon whereby cells adopt a common polarity within the tissue plane. This underlies the formation of polarized structures, for example: hairs on the skin and stereocilia in the sensory hair cells of the cochlea, as well as coordinated cell movements during gastrulation.^1,2^ However, it is best characterized in the *Drosophila* wing, where actin-rich hairs (trichomes) are oriented distally. A conserved set of six planar polarity proteins (the “core proteins”) mediates this coordinated polarization.^1–3^

**Figure 1:**
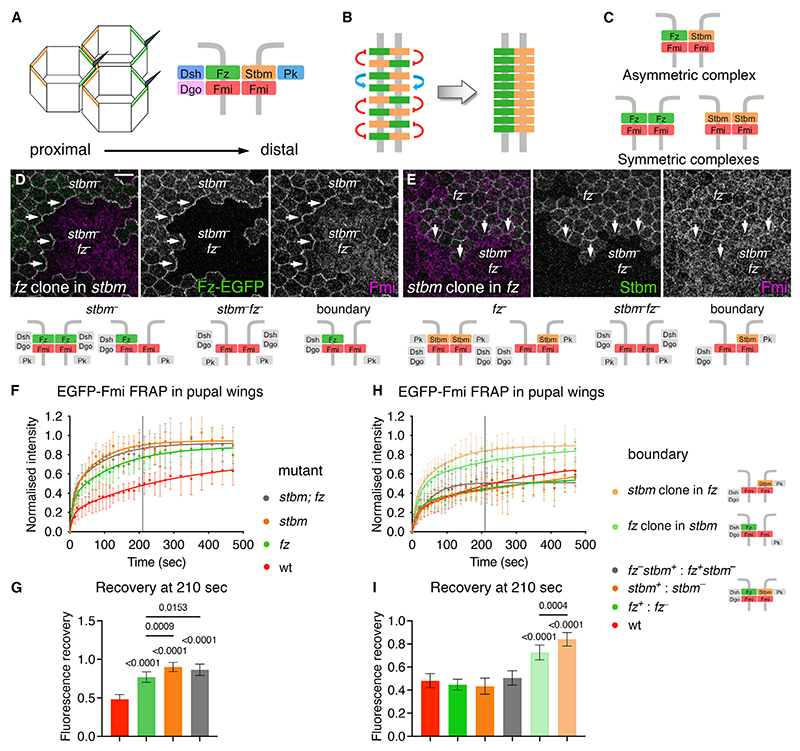
Low stability of EGFP-Fmi in asymmetric complexes lacking Fz or Stbm (A) Diagram illustrating asymmetric complex formation. Distal complexes are in green and proximal complexes are in orange (left). Right shows Fmi, Fz, Dsh, and Dgo on distal cell junctions, which interact with Fmi, Stbm, and Pk on proximal junctions of neighboring cells. (B) Diagram illustrating feedback interactions between proximal complex components (orange) and distal complex components (green). Positive interactions (blue arrows) stabilize complexes of the same orientation, and negative interactions (red) destabilize complexes of opposite orientation, leading to arrays that have a uniform orientation (right). (C) Asymmetric complex (top), with Fz on one side and Stbm on the other, and symmetric complexes (bottom), where Fz or Stbm are recruited on both sides. (D and E) 28-h after puparium formation (APF) pupal wings carrying clones of Fz-EGFP (marked by GFP immunolabeling [green]) next to *fz* mutant tissue (loss of green) in a *stbm* mutant background (D) or *stbm* clones (loss of Stbm immunolabeling [green]) in a *fz* mutant background (E). Wings immunolabeled for Fmi (magenta). Arrows point to clone boundaries, and underneath are diagrams showing the possible complexes that can form in each region of the tissue. Distal is right and anterior is up in this and all future images. Scale bar, 5 μm. (F and H) FRAP curves of EGFP-Fmi in 28-h pupal wings on proximodistal cell junctions from (F and H) wild-type (red, n = 7), (F) *fz* (green, n = 9), *stbm* (orange, n = 10), and *stbm;fz* (gray, n = 11) mutant tissues; or on (H) *fz/*+ clone boundaries (dark green, n = 10), *stbm/+* clone boundaries (dark orange, n = 9), *stbm/fz* twin clone boundaries (gray, n = 7), *fz/*+ clone boundaries in a *stbm* mutant background (pale green, n = 9), or *stbm/+* clone boundaries in a *fz* mutant background (pale orange, n = 8). Two-phase exponential curves were fitted; error bars are standard deviation (SD). Dotted line shows the time point at which intensities were compared in (G) and (I). (G and I) Fluorescence recovery at 210 s after bleaching, which is the estimated half-life of the slow phase of fluorescence recovery of wild-type EGFP-Fmi in pupal wings. Error bars are SD. Samples were compared using ANOVA with Tukey’s multiple comparisons test (G), or were compared with EGFP-Fmi in a wild-type background using ANOVA with Dunnett’s multiple comparisons test or Holm-Šídák’s test to compare the last two columns (I). See also Table S1.

The core proteins adopt asymmetric subcellular localizations across tissues that prefigure the readout of polarity, e.g., proximodistal cellular localization is observed in the *Drosophila* wing prior to emergence of trichomes. Frizzled (Fz) is a G protein-coupled receptor (GPCR) superfamily member that localizes to distal cell ends together with the cytoplasmic proteins Dishevelled (Dsh) and Diego (Dgo), while the four-pass transmembrane (TM) protein Strabismus (Stbm, also known as Van Gogh [Vang]) and the cytoplasmic protein Prickle (Pk) localize proximally. The atypical cadherin and adhesion GPCR (aGPCR) family member Flamingo (Fmi, also known as Starry night [Stan]) localizes to both proximal and distal cell ends (Figure 1A)^1–3^ and can interact homophilically in *trans* via its cadherin repeats.^4,5^ This interaction is essential for the intercellular communication that coordinates polarity between neighboring cells.^6–9^ Loss of any of the core proteins results in disruption of coordinated polarity. Importantly, all these features are conserved in vertebrate systems.^1,2,10^

The overall direction of polarity is thought to be biased by tissue-wide cues,^11,12^ while the evidence suggests that asymmetric cellular localization is a self-organizing process driven by core protein-dependent feedback interactions.^2,3^ These could be either positive, causing clustering of proximal or distal components, or negative, where proximal components destabilize distal components or vice versa (Figure 1B). Modeling suggests that such interactions in aggregate can lead to asymmetric protein localizations,^13^ and several mechanisms of positive and negative interactions have been characterized in recent years in both flies and vertebrates.^14–19^

While feedback mechanisms can explain asymmetric localization at a cellular level, how individual complexes become asymmetric at the molecular level is not understood (Figure 1C). One possibility is that complexes are preferentially asymmetric, such that Fmi *trans* dimers between neighboring cells selectively recruit Fz, Dsh, and Dgo on one side and Stbm and Pk on the other. Alternatively, symmetric or asymmetric complexes could be similarly favored, but sorting by feedback results in asymmetry at the molecular level as well as at the cellular level.^20^

Work from several labs supports the idea that there is an intrinsic asymmetry in Fmi activity that underlies the ability of complexes to become molecularly asymmetric.^8,9,21^ It was further proposed that the ability of Fmi to recruit Fz distally and Stbm proximally is a result of posttranslational modifications or conformational changes in Fmi that occur in response to its binding to one or other partner.^8,21^ However, the molecular events leading to breaking symmetry have not been further explored.

One of the difficulties in studying mechanisms of planar polarity complex assembly *in vivo* is that experiments are by necessity reductive—most simply, the effects of removing one or more proteins are assayed. However, it is more challenging to understand the minimal requirements for a process to occur. Furthermore, the feedback interactions between the core proteins mean that removal of a single component can have multiple effects—both positive and negative—on complex formation and sorting. For example, Pk can both stabilize Fz via Stbm in the neighboring cell and destabilize Fz in the same cell.^17^ We therefore decided to use a cell culture-based system to investigate the initial events in symmetry breaking and complex assembly, whereby defined core proteins can be expressed in distinct cell populations and cell mixing allows intercellular complexes to form. Such an approach has previously been used successfully to study the behavior of core proteins.^22–24^

Our results uncover the molecular mechanisms of symmetry breaking in core planar polarity protein complexes. We provide direct evidence that the initial symmetry-breaking event is the interaction between Fmi and Fz in one cell and Fmi in the neighboring cell. This initial complex is then stabilized by recruitment of Stbm on the opposite side to Fz and by recruitment of the cytoplasmic core proteins.

## Results

### Asymmetric complexes lacking either Frizzled or Strabismus are intrinsically unstable at cell junctions

We first wanted to determine how a minimally asymmetric complex forms, focusing on the three TM proteins. In particular, how does a Fmi:Fmi intercellular complex form with Fz on one side and Stbm on the other (Fz-Fmi:Fmi-Stbm, where a colon indicates an intercellular interaction and a hyphen an intracellular interaction), rather than Fz or Stbm on both sides (Fz-Fmi:Fmi-Fz or Stbm-Fmi:Fmi-Stbm) (Figure 1C)? We previously showed^21^ that Fmi is recruited to boundaries of *fz*^−^ clones in wings lacking Stbm activity (Figure 1D). Conversely, no clear accumulation of Fmi was observed on boundaries of *stbm*^−^ clones in wings lacking Fz (Figure 1E). We thus hypothesized that the accumulation of Fmi on *fz* clone boundaries represented a stable population of Fmi.

To examine this, fluorescence recovery after photobleaching (FRAP) was used to measure turnover in pupal wings of Fmi that had EGFP knocked into its extracellular region at its endogenous locus (EGFP-Fmi).^25^ FRAP of EGFP-Fmi at junctions in otherwise wild-type (WT) animals revealed a gradual increase of fluorescence after bleaching. Such recovery is the result of replacement of bleached molecules by unbleached molecules by protein turnover and is therefore a measurement of the stability of protein complexes. Recovery fitted a two-phase exponential recovery curve that was still increasing at the end of the experiment (Figure 1F, red line).

It was previously shown that loss of both Fz and Stbm causes a more severe disruption in the junctional localization of Fmi than loss of either by itself (see Figures 1D and 1E, double-mutant tissue indicated by loss of green-labeled Fz or Stbm).^21^ Using FRAP, we now find that loss of Stbm, Fz, or both significantly increases the turnover of EGFP-Fmi at cell junctions (Figure 1F, green, orange and gray lines, respectively).

We were unable to directly compare half-lives of recovery or plateaux between genotypes, as the FRAP curves were still rising, and estimates of half-lives had very wide confidence intervals. Longer FRAP experiments were not possible due to sample movement. We instead determined the proportion of recovery at a fixed time point (selected as the estimated half-life of the slow recovery phase of EGFP-Fmi under WT conditions, t = 210 s; see STAR Methods for more detail), where higher recovery indicates a higher rate of protein turnover. Recovery was significantly increased in all three mutant conditions (Figure 1G), suggesting that Fmi:Fmi complexes (lacking both Fz and Stbm) have lower stability than a full complex, as do complexes containing only Fmi and Fz or only Fmi and Stbm. However, in this experiment we cannot distinguish whether complexes missing either Fz or Stbm are symmetric or asymmetric.

We then tested the stability of asymmetric complexes where Fz or Stbm can only be present on one side of cell junctions, by examining Fmi turnover on clone boundaries. EGFP-Fmi had similar stability on boundaries of *fz*^−^ clones, *stbm*^−^ clones, and twin clones between *fz*^−^ and *stbm*^−^, where in each case asymmetric Fz-Fmi:Fmi-Stbm complexes can form (Figures 1H and 1I). In contrast, FRAP of EGFP-Fmi on boundaries of *stbm*^−^ clones in a *fz*^−^ mutant gave a significantly increased recovery of fluorescence (Figures 1H and 1I, pale orange). Thus, a Fmi:Fmi-Stbm complex is very unstable, similar to a Fmi:Fmi complex (compare with Figure 1G). Notably, EGFP-Fmi fluorescence on the boundaries of *fz*^−^ clones in *stbm*^−^ mutant tissue also showed high recovery (Figures 1H and 1I, pale green), indicating high turnover of Fz-Fmi:Fmi complexes. However, Fz-Fmi:Fmi complexes were slightly more stable than Fmi: Fmi-Stbm complexes, consistent with Fmi:Fmi-Fz complexes being favored.

### Formation of an interface between Frizzled-Flamingo in one cell and Flamingo in the neighbor is the molecular symmetry-breaking step

The small difference between fluorescent recoveries of EGFP-Fmi in Fz-Fmi:Fmi and Fmi:Fmi-Stbm complexes in pupal wings could be because cytoplasmic core proteins are still present, which might have additional roles in stabilizing/destabilizing complexes. In pupal wings, we can only remove a subset of core proteins at any one time, making it difficult to conclusively determine the minimum requirements for symmetry breaking. We therefore attempted to form intercellular complexes in a system where the protein composition could be carefully controlled. To do this, we transfected *Drosophila* S2 cells with either mEGFP- or hemagglutinin (HA)-tagged Fmi and tested whether Fmi homophilic binding could induce cell aggregation and formation of visible Fmi:Fmi interfaces (see STAR Methods) in the presence and absence of Fz and/or Stbm.

First, S2 cells transfected with Fmi-mEGFP or mEGFP-Fmi were mixed with cells transfected with Fmi-HA, and the percentage of cells showing Fmi localization on interfaces between the two cell types was scored. Cells expressing Fmi weakly aggregated, as previously reported.^4^ However, there was rarely accumulation of Fmi on cell-cell interfaces in these aggregates, and most of the Fmi localized to intracellular vesicles (Figures S1A, S1E, and S1F). However, if Fz was co-transfected with Fmi in one cell population and mixed with cells expressing only Fmi, Fmi accumulated strongly on cell boundaries (Figures S1B, S1E, and S1F). In contrast, Fmi rarely accumulated on cell interfaces between cells expressing Fmi-Stbm and cells expressing Fmi (Figures S1C, S1E, and S1F). Cells expressing Fmi-Fz formed frequent interfaces with cells expressing Fmi-Stbm (Figures S1D–S1F). Under these conditions, we saw no interfaces between cells expressing Fmi in one cell and just Fz or Stbm in the other, or between cells expressing Fz and cells expressing Stbm, in the absence of Fmi. Fz also slightly increased surface levels of Fmi in isolated cells, consistent with their ability to interact (Figures S1G–S1I).

These data show that Fmi in one cell has a strong tendency to bind Fmi-Fz in the neighboring cell but only a weak tendency to bind Fmi-Stbm. However, to break symmetry there needs to be a preference for asymmetric complexes over symmetric complexes. Notably, a significant proportion of cells expressing both Fz and Fmi formed interfaces with themselves, implying Fz-Fmi:Fmi-Fz is a favored species (Figures S1E and S1F, column 4).

We wondered whether endogenous expression of any of the core proteins might be affecting the result, as the modENCODE (https://wiki.flybase.org/wiki/FlyBase:ModENCODE_data_at_FlyBase) database indicated that some S2 cell isolates express Stbm, Fz, and Dsh. Endogenous Stbm in S2 cells may promote the formation of mixed Fz-Fmi:Fmi-Stbm interfaces in Fz-Fmi-expressing cells, rather than forming Fz-Fmi:Fmi-Fz complexes. We confirmed expression of Stbm, Fz, and Dsh in our S2 cell population, as well as in the S2R+ cell-derived line S2R+-NPT005 (Figures S1J and S1K). No expression of Fmi or Pk was detected. We do not have antibodies that can detect endogenous levels of Dgo on western blots, but Dgo was not scored as expressed in modENCODE.

**Figure 2:**
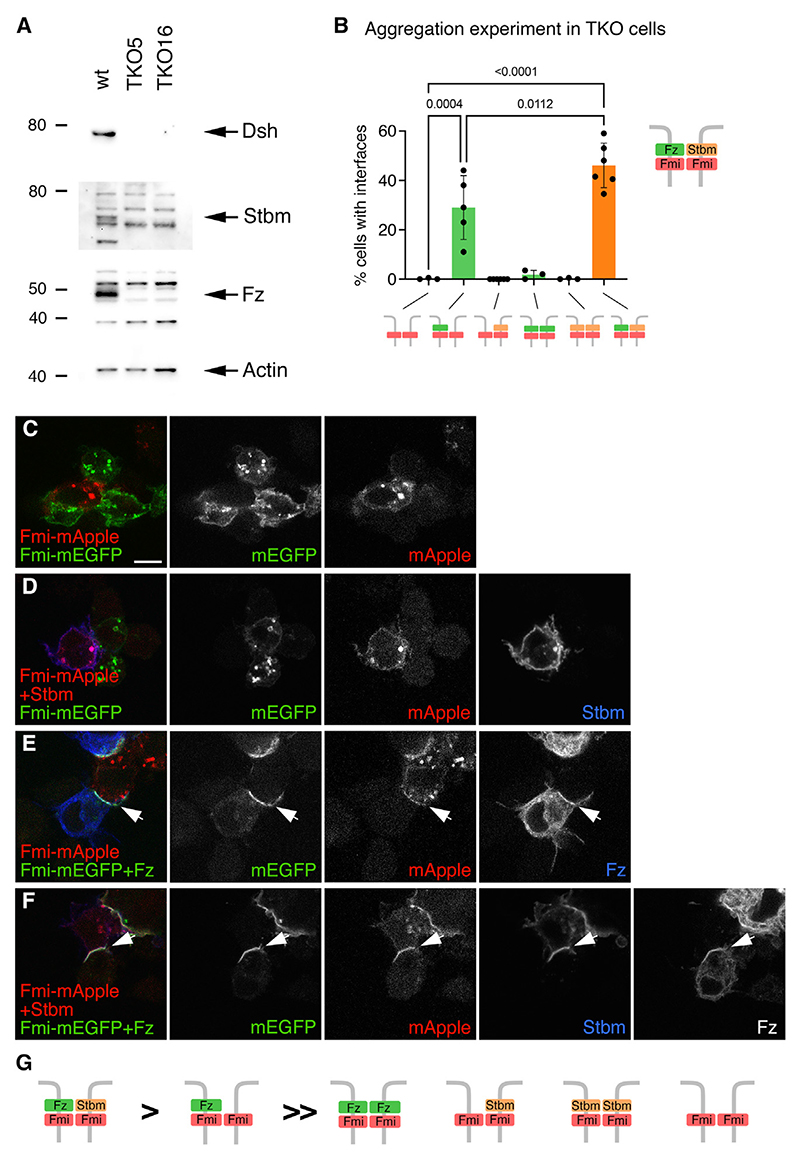
Molecular symmetry breaking by recruitment of Fz to one side of a Fmi:Fmi interface (A) Western blot on cell extracts from S2R+-NPT005 cells and two S2R+-NPT005 TKO cell lines, probed for Dsh, Stbm, Fz, or actin as loading control. Note multiple non-specific bands on blots probed for Stbm and Fz. Arrows indicate specific bands detected in wild-type but not TKO cells. (B) Quantification of Fmi:Fmi interface formation in S2R+-NPT005 TKO cells. Cells expressing Fmi-mEGFP were mixed with cells expressing Fmi-HA in the presence or absence of Fz or Stbm, as shown in the diagrams below. Graph shows the mean percentage of one cell population forming visible interfaces with the other (n = 3–6), and error bars are SD. Samples were compared using ANOVA with Tukey’s multiple comparisons test (selected p values shown). (C–F) Aggregation experiments in which S2R+-NPT005 TKO cells expressing Fmi-mEGFP (C and D) or Fmi-mEGFP and Fz (E and F) were mixed with cells expressing Fmi-mApple (C and E) or Fmi-mApple and Stbm (D and F). Cells immunolabeled for Fz (blue in E, not shown in overlay of F) or Stbm (blue in D and F) and showing EGFP (green) or mApple (red) fluorescence. Arrows point to interfaces between Fmi-mEGFP-expressing cells and Fmi-mApple-expressing cells. Scale bar, 5 μm. (G) Summary of relative strength of deduced binding interactions. See also Figure S1 and Tables S1 and S2.

CRISPR was then used to sequentially knock out expression of Dsh, Stbm, and Fz in S2R+-NPT005 cells. Loss of protein expression was confirmed by western blotting (Figure 2A). Subsequent genomic analysis revealed several lines in which there were large deletions in Dsh, Stbm, and Fz in all chromosomes (see STAR Methods), and the triple knockout (TKO) line 16 was used for subsequent studies.

We then repeated the aggregation experiments with TKO cells transfected with Fmi, Fz, and Stbm. As in S2 cells, TKO cells expressing Fmi aggregated weakly, but Fmi did not accumulate on binding interfaces (Figures 2B and 2C). The same was true for cells expressing Stbm in one or both cells (Figures 2B and 2D). However, when TKO cells co-transfected with Fmi and Fz were mixed with cells expressing Fmi, both Fmi and Fz localized to cell interfaces (Figures 2B and 2E). Finally, when cells transfected with Fmi and Fz were mixed with cells transfected with Fmi and Stbm, all three proteins localized to cell interfaces (Figures 2B and 2F).

Unlike in the S2 cells, in the TKO cells we saw almost no interfaces between cells co-expressing Fmi and Fz in both cells (Figure 2B, column 4), suggesting a strong preference for Fz-Fmi to be expressed in one cell and only Fmi in the other for interfaces to form. We surmise that endogenous Stbm in S2 cells may allow mixed Fz-Fmi:Fmi-Stbm interfaces to form, giving the impression that symmetric Fz-Fmi:Fmi-Fz complexes are present. Indeed, endogenous Stbm is recruited to interfaces of S2 cells expressing Fmi next to cells expressing Fz-Fmi but not to similar TKO cell interfaces (Figures S1L–S1O).

Overall, our data demonstrate a strong preference for Fz in just one cell to promote Fmi homophilic binding, constituting the molecular symmetry-breaking step for formation of intrinsically asymmetric complexes. Formation of the Fz-Fmi:Fmi complex then allows for recruitment of Stbm in the opposite cell, which further enhances Fmi:Fmi binding (Figures 2B and 2G).

### Strabismus and the cytoplasmic core proteins all contribute to stability of Flamingo in complexes

**Figure 3:**
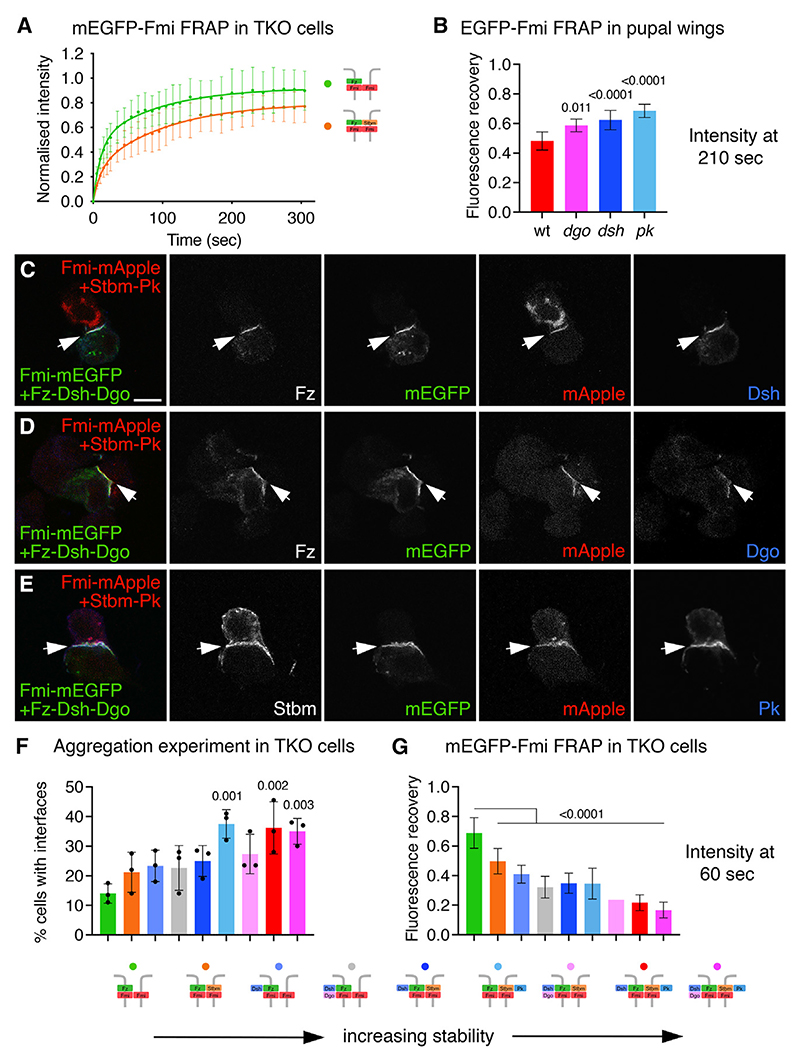
Stbm, Dsh, Pk, and Dgo additively stabilize the asymmetric complex (A) FRAP curves of mEGFP-Fmi in S2R+-NPT005 TKO cells, on interfaces with Fmi-mApple-expressing cells, in the presence of Fz in the Fmi-mApple-expressing cells (green, n = 18), or with Fz in the Fmi-mApple cells and Stbm in the mEGFP-Fmi cells (orange, n = 16). Two-phase exponential curves were fitted; error bars are SD. (B) Fluorescence recovery 210 s after bleaching during FRAP of mEGFP-Fmi on cell junctions in 28-h pupal wings from wild-type (WT) (red, n = 7), *dgo* (pink, n = 5), *dsh* (dark blue, n = 12), and *pk* (pale blue, n = 7) mutants. Error bars are SD, and samples were compared with the wild-type control using ANOVA with Dunnett’s multiple comparisons test. (C–E) Images of S2R+-NPT005 TKO cells, where cells expressing Fmi-mEGFP and Fz-T2A-Dsh-T2A-Dgo were mixed with cells expressing Fmi-mApple and Stbm-T2A-Pk. Cells show mEGFP (green) and mApple (red) fluorescence, and immunolabeled for Fz (B and C) or Stbm (D) (not shown in overlay); and for Dsh (B), Dgo (C), or Pk (D) in blue. Arrows point to interfaces between Fmi-mEGFP-expressing cells and Fmi-mApple-expressing cells. Scale bar, 5 μm. (F) Quantification of Fmi:Fmi interface formation in S2R+-NPT005 TKO cells. Cells expressing Fmi-mEGFP or Fmi-HA, and various combinations of Fz, Stbm, Pk, Dsh, and Dgo, were mixed as shown in the diagrams below. Graph shows the mean percentage Fmi-mEGFP-expressing cells forming visible interfaces with Fmi-HA-expressing cells (n = 3), and error bars are SD. Samples were compared with the left-hand column using ANOVA with Dunnett’s multiple comparisons test. (G) FRAP of mEGFP-Fmi in S2R+-NPT005 TKO cells, on interfaces with Fmi-mApple-expressing cells, with Stbm, Dsh, Pk, and/or Dgo co-transfected as indicated below. Graph shows fluorescence recovery 60 s after bleaching, which is the estimated half-life of the slow phase of fluorescence recovery of mEGFP-Fmi in Fz-Fmi:Fmi complexes in TKO cells. n = 18 (Fz-Fmi:Fmi, green), n = 16 (Fz-Fmi:Fmi-Stbm, orange), n = 18 (Dsh-Fz-Fmi:Fmi, pale blue), n = 16 (Dgo-Dsh-Fz-Fmi:Fmi, gray), n = 16 (Dsh-Fz-Fmi:Fmi-Stbm, dark blue), n = 14 (Fz-Fmi:Fmi-Stbm-Pk, cyan), n = 13 (Dgo-Dsh-Fz-Fmi:Fmi-Stbm, pale pink), n = 15 (Dsh-Fz-Fmi:Fmi-Stbm-Pk, red), and n = 15 (Dgo-Dsh-Fz-Fmi:Fmi-Stbm-Pk, dark pink). Error bars are SD, and samples were compared with the wild-type control using ANOVA with Dunnett’s multiple comparisons test. Note that SD could not be determined for one sample (Dgo-Dsh-Fz-Fmi:Fmi-Stbm), due to poor curve fitting. Despite varying expression levels, we found no effect of differing fluorescence intensity on degree of fluorescence recovery within a genotype. See also Figure S2 and Tables S1 and S2.

EGFP-Fmi accumulates on the boundaries of *fz* clones in pupal wings lacking Stbm activity, but it has low stability (Figures 1H and 1I). This could be due to negative feedback caused by the presence of cytoplasmic core proteins or reflect the intrinsic instability of Fz-Fmi:Fmi complexes. We thus investigated the stability of Fmi in TKO cells, where no cytoplasmic core proteins are present, when cells expressing mEGFP-Fmi were mixed with cells expressing Fmi-mApple and Fz. FRAP of mEGFP-Fmi on boundaries where mEGFP-Fmi and Fmi-mApple co-localized showed that complexes were intrinsically unstable, with 90% recovery by the end of the experiment (Figure 3A). mEGFP-Fmi stability was increased on the boundaries of cells expressing mEGFP-Fmi and Stbm, next to cells expressing Fmi-mApple and Fz (Figure 3A).

Previous *in vivo* results have suggested that the cytoplasmic proteins have overall stabilizing effects on Fz and Stbm.^17,26–28^ FRAP of EGFP-Fmi was carried out in pupal wings lacking either Pk, Dsh, or Dgo. In each case, a decrease in stability was observed (Figures 3B and S2A), indicating that the cytoplasmic core proteins also have a net positive effect on EGFP-Fmi stability *in vivo*. However, the contribution of each protein to overall stability cannot be determined, as negative effects may also be occurring.

We next determined the effect of the cytoplasmic core proteins on Fmi:Fmi interface formation in TKO cells. We used the T2A system to co-express either distal complex components (Dsh and/or Dgo with Fz) or proximal complex components (Stbm and Pk). Western blotting confirmed efficient cleavage at the T2A site (Figures S2B–S2E), and immunolabeling showed recruitment of Dsh, Dgo, and Pk to cell-cell interfaces in aggregation experiments with Fmi, Fz, and Stbm (Figures 3C–3E). Recruitment of Dsh and Dgo was dependent on co-expression of Fz, and recruitment of Pk was dependent on co-expression of Stbm.

Cells expressing Fz-Fmi and cells expressing Fmi or Fmi-Stbm formed more frequent interfaces when Dsh or Dsh and Dgo were co-expressed with Fz and/or Pk was co-expressed with Stbm (Figure 3F). As a more sensitive assay, we used FRAP to analyze how the addition of cytoplasmic core proteins affected stability of complexes. We compared recovery at 60 s, which is the estimated half-life of mEGFP-Fmi recovery in Fz-Fmi:Fmi complexes. Although expression levels varied between different transfected cells, importantly we found no effect of varying expression on degree of fluorescence recovery. Notably, FRAP of mEGFP-Fmi showed an increase in Fmi stability at cell interfaces as the number of core proteins co-expressed is increased, with Fmi stability being strongest in cells expressing three or four proteins in addition to Fz-Fmi:Fmi (Figures 3G and S2F). This confirms that Stbm and the three cytoplasmic core proteins all contribute to complex stability.

### Reconstituted complexes do not sort into domains of opposite polarity

In our cell culture experiments thus far, cells expressing distal complex components were aggregated with cells expressing proximal complex components. This excludes the possibility of negative feedback interactions between distal and proximal core proteins. We next expressed all complex components in the same cell population to see if this would lead to negative feedback interactions able to locally destabilize oppositely oriented complexes and whether this would be sufficient to promote segregation of distal and proximal complex components. We could envisage two levels of segregation: first, local sorting of complexes, whereby complexes within an interface segregated into discrete domains; second, cell-scale sorting, whereby complexes segregate to opposite cell ends.

**Figure 4:**
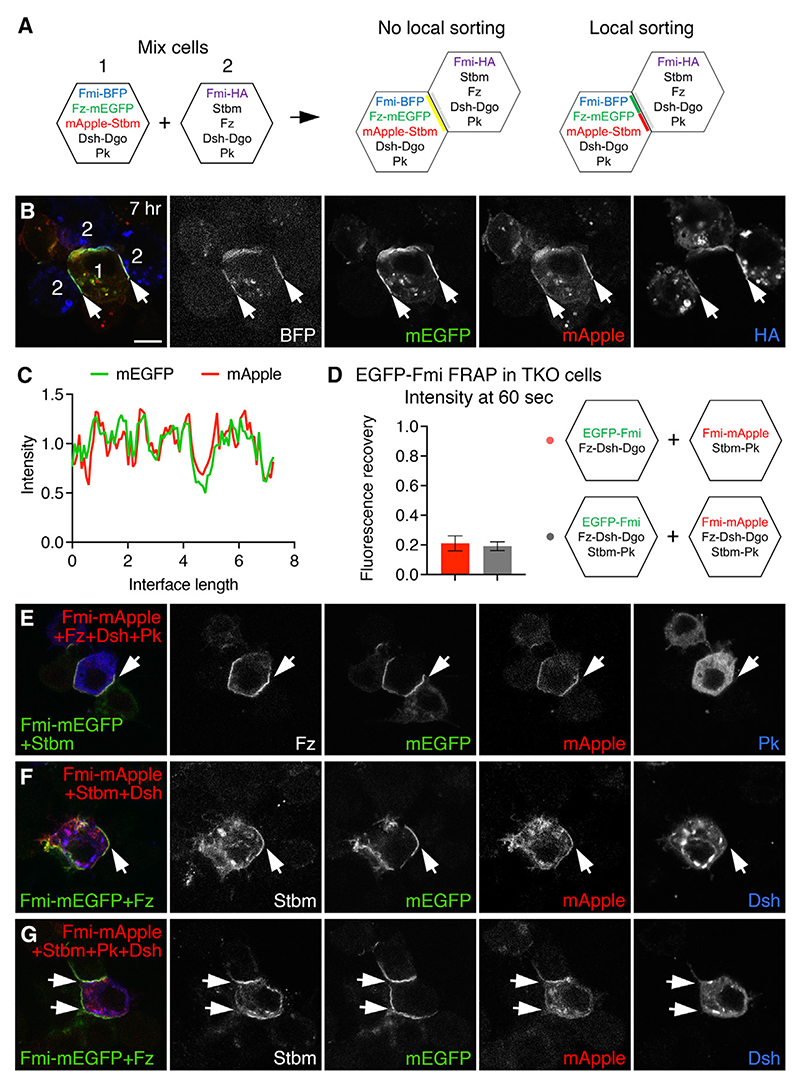
Lack of complex sorting in aggregated cells (A) Schematic of the experiment in (B) and (C). Cells expressing the combinations of proteins shown are allowed to form aggregates. Fz-mEGFP will form complexes with untagged Stbm in the neighboring cell, and mApple-Stbm will form interfaces with untagged Fz in the neighboring cell. If there is no local sorting within an interface, then Fz-mEGFP and mApple-Stbm will distribute evenly along the interface. If there is local sorting, then Fz-mEGFP and mApple-Stbm would be expected to segregate into discrete domains. (B) S2R+-NPT005 TKO cells as described in (A) mixed and allowed to interact for 7 h. Cells immunolabeled for HA (blue) and showing BFP (not in overlay) and mEGFP (green) and mApple (red) fluorescence. Arrows point to typical interfaces between the two cell types, showing co-localization along the entire interface. Scale bar, 5 μm. (C) Line scan along a typical interface from the experiment in (B), showing co-localization of peaks of Fz-mEGFP and mApple-Stbm fluorescence. The ratio of mEGFP to mApple fluorescence was compared pixel-by-pixel along the whole interface and differed from the mean with an SD of 14.3%. Over 18 wings, the average SD in the mEGFP-mApple ratio compared with the mean was 15.9%. (D) Fluorescence recovery 60 s after bleaching during FRAP of mEGFP-Fmi in S2R+-NPT005 TKO cells, on interfaces with Fmi-mApple-expressing cells. Red bar shows interfaces between cells expressing mEGFP-Fmi, Fz, Dsh, and Dgo mixed with cells expressing Fmi-mApple, Stbm, and Pk (n =7); gray bar shows interfaces between cells expressing mEGFP-Fmi and cells expressing mApple-Fmi, where both cell types co-express Fz, Dsh, Dgo, Stbm, and Pk (n = 7). Error bars are SD, and samples were compared with an unpaired t test; no significant difference was seen. (E) S2R+-NPT005 TKO cells expressing Fmi-mEGFP and Stbm were mixed with cells expressing Fmi-mApple and Fz-T2A-Dsh-T2A-Pk. Cells show mEGFP (green) and mApple (red) fluorescence, and immunolabeled for Fz (not shown in overlay) and Pk (blue). Arrows point to interfaces between Fmi-mEGFP-expressing cells and Fmi-mApple-expressing cells, and Pk is not recruited to interfaces. (F and G) S2R+-NPT005 TKO cells expressing Fmi-mEGFP and Fz were mixed with cells expressing Fmi-mApple and Stbm-T2A-Dsh (F) or Stbm-T2A-Dsh-T2A-Pk (G). Cells show mEGFP (green) and mApple (red) fluorescence, and immunolabeled for Stbm (not shown in overlay) and Dsh (blue). Arrows point to interfaces between Fmi-mEGFP-expressing cells and Fmi-mApple-expressing cells, and Dsh is not recruited to interfaces. See also Figure S3 and Tables S1 and S2.

Due to variable transfection efficiencies leading to neighboring cells expressing different protein levels, it was not possible to determine if cell-scale sorting occurred. However, we were able to test for local sorting by co-transfecting one cell population with a full set of core proteins with Fz-mEGFP and mApple-Stbm and transfecting the other population with untagged proteins. We expected complexes to form on interfaces with Fz-mEGFP in one cell and untagged Stbm in neighboring cells and with mApple-Stbm in one cell and untagged Fz in the neighbor. If there was local sorting of complexes along an interface, then Fz-mEGFP and mApple-Stbm might segregate into separate domains, whereas if there was no local sorting, then complete co-localization of Fz-EGFP and mApple-Stbm along all interfaces would be expected (Figure 4A). We allowed cells to interact for either 7 or 24 h consistent with the known time frame of polarization *in vivo*,^7,29,30^ and in both cases, no local sorting was observed (Figures 4B and S3A), and there was a high degree of co-localization along interfaces (Figure 4C). Therefore, distal complexes and proximal complexes do not visibly segregate within an interface.

To further test for negative feedback interactions, we measured the stability of mEGFP-Fmi in complexes, comparing a situation where cells expressing distal complex components were apposed to cells expressing proximal complex components, to one where all cells expressed both proximal and distal complex components. mEGFP-Fmi fluorescence recovered similarly in both cases (Figures 4D and S3B), suggesting that the presence of complexes in both orientations had negligible effects on complex stability. We conclude that there is no evidence for negative feedback interactions between proximal and distal complex components in our S2 cell system.

The distal component Dsh binds to both the proximal components Stbm and Pk *in vitro*, and it has been suggested that this binding may be necessary for feedback.^17,31–36^ We therefore asked whether, in our cell aggregation system, proximal components can be recruited into distal complexes or vice versa. When TKO cells co-expressing Pk with Fmi-Fz-Dsh were mixed with cells expressing Fmi-Stbm, no recruitment of Pk to interfaces was seen, unlike when Pk is co-expressed with Fmi-Stbm (Figure 4E, compare with Figure 3E). Similarly, there was no recruitment of Dsh into Fmi-Stbm or Fmi-Stbm-Pk “proximal” complexes (Figures 4F and 4G, compare with Figure 3C). Endogenous Dsh in S2 cells was also recruited to interfaces in cells expressing Fmi-Fz but not when they expressed Stbm or Stbm-Pk (Figures S3C–S3J). Likewise, endogenous Stbm in S2 cells was recruited only on the opposite side to Fmi-Fz-Dsh (Figures S3K–S3N). This suggests that any *in vivo* binding interactions between Dsh and Stbm or Pk are at best transient.

### Flamingo cadherin repeats require Frizzled for their homophilic *trans* interaction

**Figure 5:**
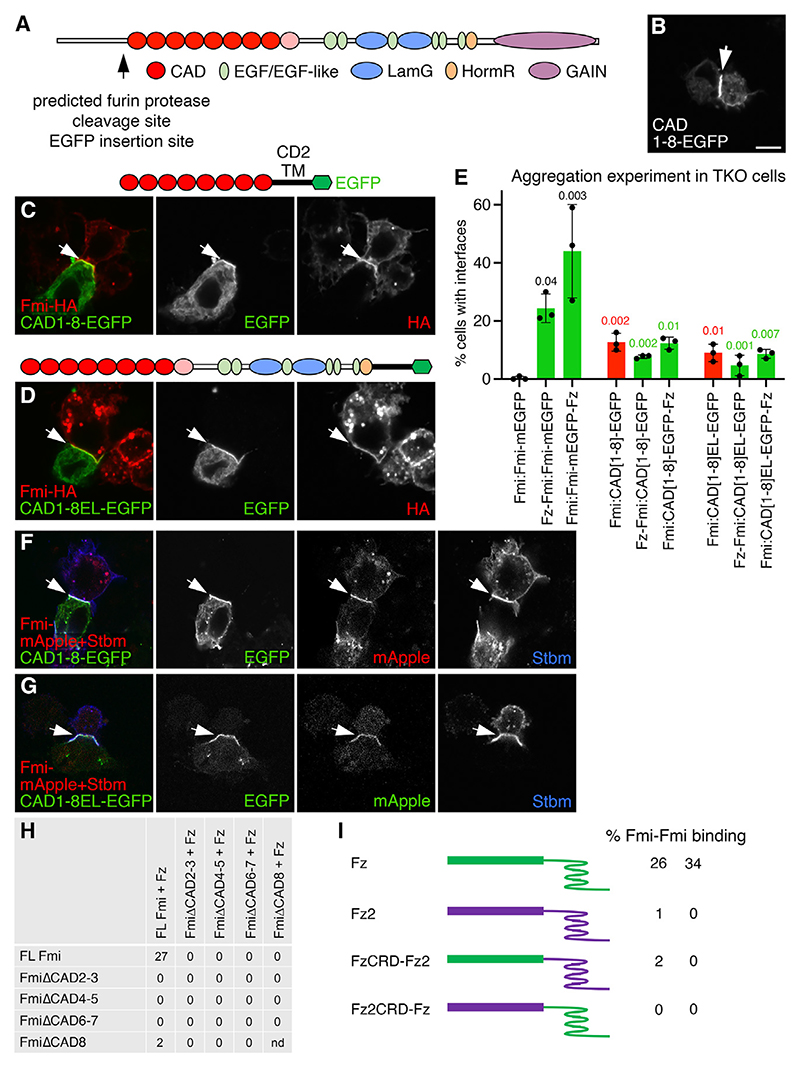
Interaction of the cadherin domains of Fmi (A) Schematic of the extracellular region of Fmi. An N-terminal EGFP tag was inserted immediately after the predicted furin protease site.^5,39^ (B) S2R+-NPT005 TKO cells expressing Fmi[CAD1–8]-CD2TM-EGFP, immunolabeled for GFP. Arrows point to interfaces between two cells expressing Fmi [CAD1–8]. Scale bar, 5 μm. (C and D) S2R+-NPT005 TKO cells expressing Fmi [CAD1–8]-CD2TM-EGFP (C) or Fmi[CAD1–8]-EL-CD2TM-EGFP (D), as illustrated in the diagrams above, mixed with cells expressing HA-tagged Fmi. Cells immunolabeled for GFP (green) and HA (red). Arrows point to interfaces between the two cell types. (E) Quantification of Fmi:Fmi interface formation in S2R+-NPT005 TKO cells. Cells expressing Fmi-mEGFP, CD2Sig-Fmi[CAD1–8]-CD2TM-EGFP, or Fmi[CAD1–8]-EL-CD2TM-EGFP were mixed with cells expressing Fmi-HA, in the presence or absence of Fz in one or other cell type. Graph shows the mean percentage Fmi-mEGFP-expressing cells forming visible interfaces with Fmi-HA-expressing cells (n = 3), and error bars are SD. Samples were compared using ANOVA with Dunnett’s multiple comparisons test, where values in black are comparisons to full-length Fmi, and values in red and green are comparisons to the equivalent Fmi-mEGFP data. (F and G) S2R+-NPT005 TKO cells expressing Fmi [CAD1–8]-CD2TM-EGFP (F) or Fmi[CAD1–8]-EL-CD2TM-EGFP (G), mixed with cells expressing Fmi-HA and Stbm. Cells showing EGFP (green) and mApple (red) fluorescence, and immunolabeled for Stbm (blue). Arrows point to interfaces between the two cell types. (H) Table showing percentage of cells with interfaces in S2 cells expressing either wild-type Fmi-EGFP, or Fmi-EGFP in which various cadherin domains are deleted (see Figure S4D), in the presence or absence of Fz in one or other cell type. (I) Schematic of chimeras between Fz and DFz2, in which the CRDs are swapped, and percentage of cells with interfaces in two aggregation experiments. Fz promotes binding of Fmi-HA to Fmi-mEGFP in neighboring cells, whereas DFz2 and the chimeric molecules do not. See also Figures S4 and S5 and Tables S1 and S2.

Our results so far indicate that Fz interacts with Fmi to promote homophilic interactions. We therefore wanted to understand how Fz could affect Fmi binding by dissecting the role of the extracellular domain of Fmi. In addition to its cadherin repeats, Fmi contains several epidermal growth factor (EGF) or EGF-like repeats, two laminin G (LamG) domains, a hormone receptor (HormR) domain, and a GPCR autoproteolysis-inducing (GAIN) domain (Figure 5A).^37,38^

One of the vertebrate homologs of Fmi, Celsr2, was recently reported to mediate homophilic adhesion via a head-to-tail arrangement of its first eight cadherin domains.^5^ We tested whether the cadherin domains of Fmi (CAD1–8) were sufficient to interact homophilically, by making a heterologous fusion with the signal sequence and TM domains of the immunoglobulin superfamily member CD2. CAD1–8 mediated *trans* interactions with itself (Figure 5B) and to full-length Fmi in neighboring cells (Figure 5C). In contrast, expression of fewer cadherin repeats (CAD1, CAD1–2, and CAD1–4) was not sufficient for binding, even though they localized well to the cell surface (Figures S4A–S4C).

Notably, CAD1–8 can bind weakly to full-length Fmi in neighboring cells when isolated from the rest of the Fmi molecule, but full-length Fmi cannot robustly interact with itself in the absence of Fz (Figures 2B, 2C, and 5E, compare columns 1 and 4). However, binding of CAD1–8 to full-length Fmi was less than binding of full-length Fmi and Fz to Fmi-mEGFP in neighboring cells (Figure 5E, compare columns 2 and 4). A longer molecule that included the EGF repeats, the LamG domains, and the HormR domain (CAD1–8EL) bound to full-length Fmi similarly to CAD1–8 (Figures 5D and 5E, compare columns 4 and 7). Furthermore, expression of Fz in either the CAD1–8-expressing cell or the cell expressing full-length Fmi did not enhance the interaction (Figure 5E, compare column 4 with columns 5 and 6 and column 7 with columns 8 and 9), and both CAD1–8 and CAD1–8EL, in the absence of Fz, promote recruitment of Stbm by full-length Fmi in the neighboring cell (Figures 5F and 5G).

Thus, we find that CAD1–8 can bind full-length Fmi in a Fz-independent manner, whereas normally Fz is required on one side of the Fmi:Fmi dimer for robust interaction. To explain this, we tentatively propose that Fmi is normally in a “closed” conformation that only allows weak/transient homophilic *trans* binding. Expression of Fz in one cell “opens” Fmi and allows for robust binding of Fmi to itself in *trans*. Expression of CAD1–8 in isolation mimics the action of Fz in opening Fmi in one cell, and this can both interact with full-length Fmi in its neighbor and cause it to recruit Stbm (see discussion).

We next tested whether deletion of any of the cadherin domains of Fmi affected its ability to interact with Fmi and Fz in the neighboring cells. Deletion of any pair of cadherin domains (ΔCAD2-3, ΔCAD4-5, or ΔCAD6-7; see Figure S4D) completely abrogated aggregation of cells with cells expressing full-length Fmi and Fz (Figure 5H), even though they localized to the cell surface similarly to full-length Fmi (Figures S4E–S4K). When in *cis* with Fz, these deletions also failed to bind to full-length Fmi in neighboring cells, and no combination of deleted molecules could bind each other (Figure 5H). Deletion of CAD8 alone had a milder effect on the ability of Fmi to bind Fmi-Fz in *trans* (Figures 5H and S4L). Therefore, all cadherin repeats contribute to the homophilic *trans* binding of Fmi.

### The Frizzled CRD is necessary but not sufficient for promotion of Flamingo homophilic *trans* interactions

Previous work has suggested that deletion of the first LamG domain of Celsr3 blocks its binding to Fz.^40^ We tested the effects of deleting the EGF and LamG domains of Fmi (Figure S4D) on its interaction with full-length Fmi, either with Fz in the same cell or the opposite cell.

Deletion of both LamG domains, or of LamG1 alone, completely abrogated *trans* interactions, regardless of which cell expressed Fz, whereas deletion of LamG2 had no effect (Figures S5A and S5C). However, FmiΔLamG and FmiΔLamG1 failed to localize to the cell surface (Figures S5D and S5F–S5I). Interestingly, deleting all EGF and LamG repeats of Fmi did not completely block its localization to the cell surface (Figures S5E and S5I), and weak binding was observed to full-length Fmi-Fz in neighboring cells (Figures S5B and S5C). However, no binding to full-length Fmi was observed when Fz was in the same cell as FmiΔEGFΔLamG (Figure S5C), which may indicate a role for these domains in interacting with Fz.

We then tested whether the cysteine-rich domain (CRD) of Fz was important for the ability of Fz to stabilize Fmi:Fmi *trans* interactions. Chimeras between Fz and its ortholog DFz2 were generated by swapping their CRDs. DFz2 is active in canonical Wingless signaling but has no known role in planar polarity, and as expected, it does not promote Fmi:Fmi *trans* interactions in our aggregation assay in TKO cells (Figure 5I). Molecules carrying the Fz CRD fused to the Fz2 TM domain and cytoplasmic tail, and molecules carrying the Fz2 CRD fused to the Fz TM domain and cytoplasmic tail both failed to enhance Fmi:Fmi *trans* interactions (Figure 5I), even though all molecules localize at the cell surface (Figures S5J–S5N). We therefore conclude that the Fz CRD is necessary but not sufficient for promoting *trans* interactions between Fmi molecules in neighboring cells.

### The intracellular loops of Flamingo are required for its stabilization by Strabismus

Our final question was how binding of Fz to Fmi in one cell allows Fmi to bind to Stbm in its neighbor. Our assumption is that *trans* Fmi binding causes a conformational change in Fmi that exposes a Stbm binding interface. As a first step to understanding this, we asked what regions of Fmi are required to interact with Stbm.

**Figure 6:**
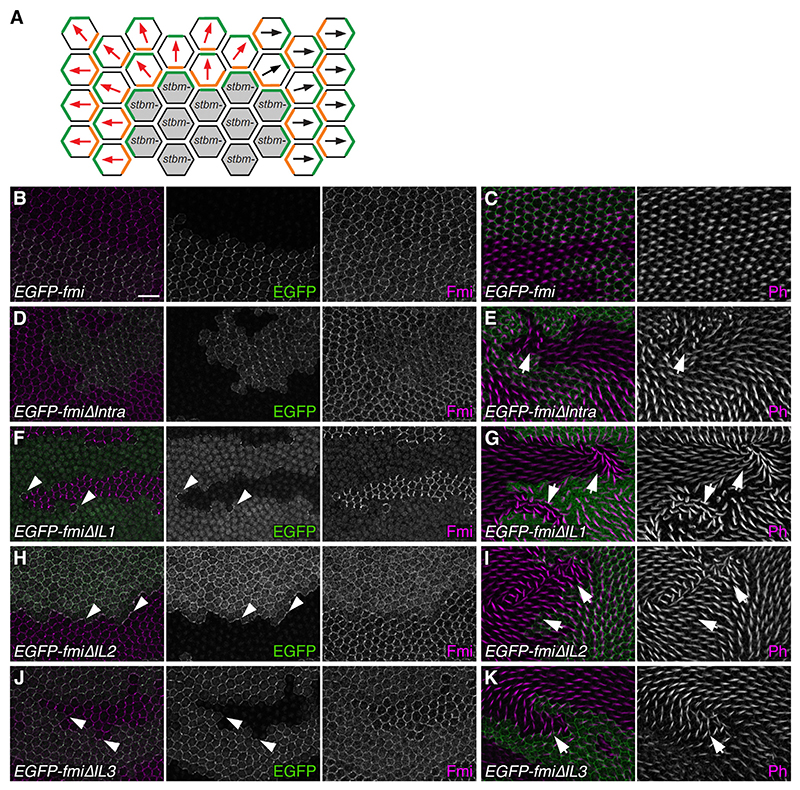
Deletion of intracellular loops of Fmi phenocopies loss of Stbm (A) Diagram showing proximal non-autonomy around *stbm* mutant clones. In the boundary cells lacking Stbm, the Fz localizes to the clone boundary, where it can form complexes with Stbm in the neighboring wild-type cells. This reverses the orientation of complexes on the proximal side of the clone and causes trichomes to point away from the clone (red arrows), rather than distally (black arrows). (B–K) Pupal wings carrying clones of *EGFP-fmi* variants, juxtaposed to wild-type tissue. (B and C) Wild-type *EGFP-fmi*, (D and E) *EGFP-fmiΔIntra*, (F and G) *EGFP-fmiΔIL1*, (H and I) *EGFP-fmiΔIL2*, and (J and K) *EGFP-fmiΔIL3*. (B, D, F, H, and J) 28-h APF wings immunolabeled for Fmi (magenta) and showing EGFP fluorescence (green). (C, E, G, I, and K) Wings from flies raised at 29°C for 27 h 15 min, immunolabeled for GFP (green) and labeled for phalloidin (magenta). Arrowheads point to accumulation of EGFP-Fmi on clone boundaries. Arrows point to non-autonomous trichome swirling, in wild-type tissue on the distal side of *fmiΔIntra* clones (E) and the proximal side of *fmiΔIL1, fmiΔIL2* and *fmiΔIL3* clones (G, I, and K). Scale bar, 10 μm. See also Figure S6.

We previously showed that a form of Fmi lacking most of its cytoplasmic tail (FmiΔIntra) preferentially localized to distal cell edges together with Fz, rather than to proximal cell edges.^21^ Mild proximal non-autonomy was also seen on the boundaries of FmiΔIntra clones, and core proteins accumulated on clone boundaries. This is the same phenomenon that is seen on the boundaries of *stbm* mutant clones (Figure 6A) and is consistent with FmiΔIntra having a reduced interaction with Stbm.

As the original FmiΔIntra molecule was expressed under a heterologous *Actin5C* promoter, we made a new FmiΔIntra version expressed under the endogenous *fmi* promoter, and with an N-terminal EGFP tag rather than a C-terminal EGFP (*EGFP-fmi* rescue construct; see STAR Methods). In EGFP-FmiΔIntra, amino acids 3,087–3,529 were deleted, retaining the last C-terminal 74 amino acids, so as to maintain the PDZ-binding motif (PBM) that is required for Snx27-dependent recycling of Fmi.^25^ In pupal wing clones, full-length EGFP-Fmi localized asymmetrically to junctions, at similar levels to endogenous Fmi (Figure 6B). Fz and Stbm recruitment was similar to that of endogenous Fmi, and trichome polarity was normal (Figures 6C, S6A, and S6B). EGFP-FmiΔIntra was expressed at reduced levels and recruited reduced levels of Fz and Stbm (Figures 6D, S6C–S6E, and S6V). Surprisingly, it still localized asymmetrically, with similar levels of asymmetry to full-length Fmi (Figures 6D, S6T, and S6U). No proximal non-autonomy was observed next to EGFP-FmiΔIntra clones, and instead there was occasional mild distal non-auton-omy (Figure 6E). A version of EGFP-FmiΔIntra that also lacks the PBM (EGFP-FmiΔIntraΔPBM) was expressed at even lower levels, presumably because of reduced recycling, but also showed mild distal non-autonomy (Figures S6F–S6J and S6T–S6V). This suggests that the C-terminally abridged Fmi does not interact preferentially with Fz, as we previously thought, and the discrepancy may be because of expression levels or the position of the EGFP tag (see discussion).

Fmi is a member of the aGPCR family, and activation of such molecules by ligand binding may cause conformational changes in the seven-pass TM region, leading to the activation of intracellular signaling cascades.^41,42^ We therefore tested if any of the three intracellular loops (IL) of Fmi are required for its interaction with Stbm. We used recombineering on the EGFP-Fmi rescue construct to replace 6 amino acids in each loop with 24 amino acids partly encoded by an inserted flippase recognition target (FRT) site (a by-product of the recombineering procedure). In pupal wing clones, EGFP-FmiΔIL1 localized poorly to the cell surface, recruited Fz and Stbm poorly, and there was little core protein asymmetry (Figures 6F, S6K–S6M, and S6T–S6V). In spite of this, junctional complexes were seen at clone boundaries (Figure 6F), and there was robust proximal non-autonomy (arrows in Figure 6G). This suggests that the small amount of EGFP-FmiΔIL1 that localizes to the cell surface interacts preferentially with Fz rather than Stbm. EGFP-FmiΔIL2 is better recruited to cell junctions than EGFP-Fmi but still has poor asymmetry (Figures 6H, S6N–S6P, and S6T–S6V), whereas EGFP-FmiΔIL3 is recruited even better and retains some asymmetric localization (Figures 6J and S6Q–S6V). Notably, there is accumulation of Fmi on both ΔIL2 and ΔIL3 clone boundaries and proximal non-autonomy that was more consistent in EGFP-FmiΔIL2 than EGFP-FmiΔIL3 (Figures 6H–6K).

**Figure 7:**
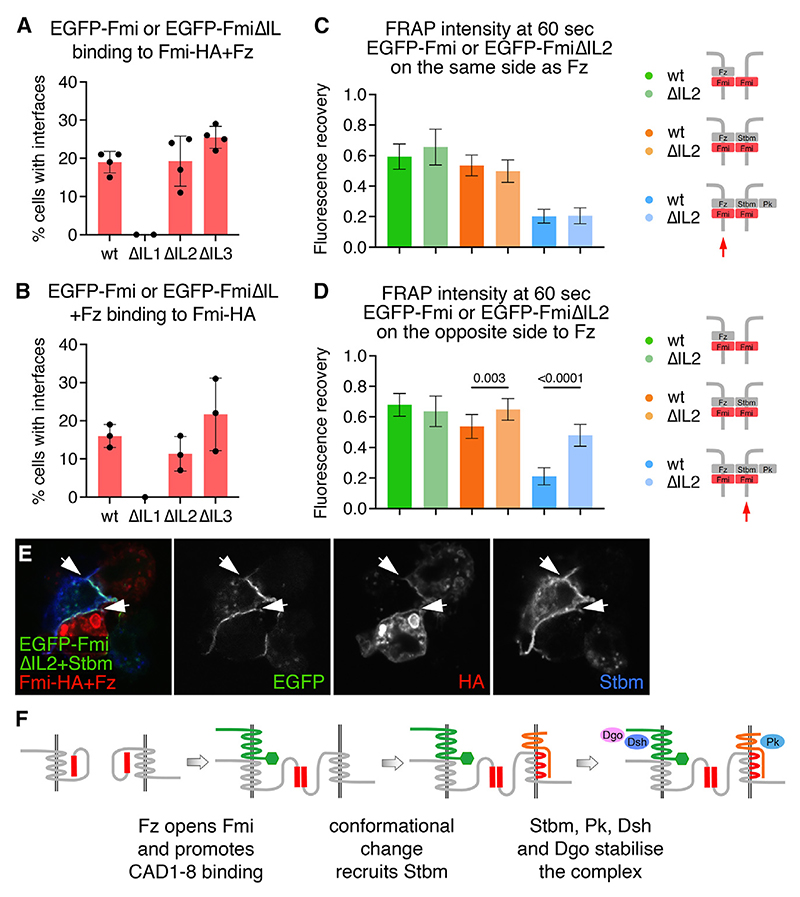
The intracellular loops of Fmi are required for it to be stabilized by Stbm (A and B) Graphs showing the mean percentage of S2R+-NPT005 TKO cells expressing intracellular loop mutations of Fmi forming visible interfaces with Fmi-HA-expressing cells, in the presence or absence of Fz in one or other cell. (A) n = 4 except for ΔIL1, n = 2. (B) n = 3, except for ΔIL1, n = 1. Error bars are SD. Samples were compared with EGFP-Fmi using ANOVA with Dunnett’s multiple comparisons test (note that ΔIL1 had too few replicates for ANOVA). (C and D) Fluorescence recovery 60 s after bleaching during FRAP of EGFP-Fmi (darker bars) or EGFP-FmiΔIL2 (paler bars) in S2R+-NPT005 TKO cells, on interfaces with Fmi-mApple-expressing cells. Green bars show FRAP on Fmi:Fmi interfaces, where the Fz is on the same side (C) or the opposite side (D) to EGFP-Fmi or EGFP-FmiΔIL2 (see red arrows in diagrams to the right). Orange bars show FRAP on Fz-Fmi:Fmi-Stbm interfaces, and blue bars show FRAP on Fz-Fmi:Fmi-Stbm-Pk interfaces. (C) n = 11 (dark green), n = 9 (pale green), n = 10 (dark orange), n = 11 (pale orange), n = 9 (dark blue), and n = 11 (pale blue). (D) n = 11 (dark green), n = 10 (pale green), n = 11 (dark orange), n = 11 (pale orange), n = 9 (dark blue), and n = 10 (pale blue). Error bars are SD; pairs of samples were compared using ANOVA with Holm-Šídák’s multiple comparison test. (E) S2R+-NPT005 TKO cells expressing EGFP-FmiΔIL2 and Stbm were mixed with cells expressing Fmi-HA and Fz. Cells immunolabeled for HA (red) or Stbm (blue) and showing EGFP fluorescence (green). Arrows point to interfaces between EGFP-Fmi-expressing cells and Fmi-HA-expressing cells. Scale bar, 5 μm. (F) Model for assembly of an asymmetric complex. See also Figure S7 and Tables S1 and S2.

These results suggest that mutations in the three intracellular loops of Fmi affect its interaction with Stbm to varying degrees. To confirm this, we made use of our cell culture aggregation assay. We first expressed each Fmi variant in TKO cells and tested whether the variants supported formation of Fmi:Fmi interfaces. EGFP-FmiΔIL2 and EGFP-FmiΔIL3 both formed interfaces with full-length Fmi, similarly to WT EGFP-Fmi, either when Fz was in the same cell (Fz-FmiΔIL:Fmi) or in the opposite cell (Fz-Fmi:FmiΔIL) (Figures 7A, 7B, and S7A–S7C). EGFP-FmiΔIL1 failed to form any interfaces (Figures 7A and 7B), and extracellular staining revealed that it reached the cell surface poorly, as in pupal wings (Figures S7D–S7H). We conclude that FmiΔIL2 and FmiΔIL3 have no defect in Fmi:Fmi *trans* interactions, and they interact normally with Fz.

We then used FRAP to test whether Stbm could stabilize complexes containing EGFP-FmiΔIL2, either in the same cell or the opposite cell. In the absence of Stbm, EGFP-FmiΔIL2 had similar stability to full-length EGFP-Fmi, regardless of whether Fz is in the same cell as FmiΔIL2 (Fz-FmiΔIL2:Fmi) or the opposite cell (Fz-Fmi:FmiΔIL2) (green in Figures 7C, 7D, S7I, and S7J). The stability of EGFP-FmiΔIL2 was increased by expression of Fmi-Stbm or Fmi-Stbm-Pk in the opposite cell, similar to WT Fmi (Fz-FmiΔIL2:Fmi-Stbm and Fz-FmiΔIL2:Fmi-Stbm-Pk, orange and blue, respectively, in Figures 7C and S7I). However, Stbm failed to increase EGFP-FmiΔIL2 stability, unlike WT Fmi, when it was expressed in the same cell (Fz-Fmi:FmiΔIL2-Stbm, orange in Figures 7D and S7J). Expression of Stbm-Pk in the same cell as EGFP-FmiΔIL2 (Fz-Fmi:FmiΔIL2-Stbm-Pk) stabilized it slightly, but the increase in stability was much less than for WT EGFP-Fmi (blue in Figures 7D and S7J). We wondered whether this reflected a failure of Stbm to be recruited into intercellular complexes by FmiΔIL2, but immunolabeling showed that Stbm was recruited by FmiΔIL2, similarly to WT Fmi (Figure 7E). This suggests that mutation of IL2 does not prevent the binding of Stbm to Fmi, but it does affect the ability of the bound Stbm to stabilize Fmi.

Finally, we tested the ability of EGFP-FmiΔIL3 to stabilize complexes. Interestingly, EGFP-FmiΔIL3 recruited Stbm and was stabilized by Stbm and Pk, even when they were expressed in the same cell (Figures S7K–S7M). We propose that, consistent with the weaker proximal non-autonomy of EGFP-FmiΔIL3 compared with EGFP-FmiΔIL2, the ΔIL3 variant has sufficient activity to be stabilized by Stbm in the cell culture assay.

Overall our results suggest that the TM region of Fmi plays a critical role in the ability of Stbm to stabilize Fz-Fmi:Fmi complexes.

## Discussion

In this work, we have attempted to understand the sequence of events leading to the molecular asymmetry of individual core planar polarity protein complexes. We provide evidence that planar polarity complexes are intrinsically asymmetric. We demonstrate that binding of Fz to Fmi in one cell stabilizes homophilic intercellular Fmi:Fmi interactions and that this is the key event in breaking symmetry. Binding of Fz on one side of the complex then promotes recruitment of Stbm to the opposite side of the complex. Recruitment of Stbm and the cytoplasmic core proteins into the complex is necessary to stabilize Fmi at intercellular interfaces (Figure 7F).

Our previous experiments in pupal wings^21^ showed that Fmi is visibly recruited to interfaces between cells expressing Fmi and those expressing Fmi and Fz. In contrast, Fmi is not strongly recruited to interfaces between cells expressing Fmi and those expressing Fmi and Stbm. Using FRAP, we now show that this difference reflects only a small increase in Fmi stability at junctions. To further understand this, we developed a cell-based system. Using this, we can reconstruct complex formation between specific subsets of components, without the complication of negative feedback interactions. In this system, the result is unambiguous: interfaces never form between cells expressing only Fmi, or cells expressing Fmi and Stbm, while there is a strong preference for Fmi:Fmi interfaces to form when Fz is in only one cell.

How does Fz in one cell stabilize Fmi homophilic *trans* interactions? It was recently shown by high-speed atomic force microscopy experiments that the first eight cadherin repeats of the vertebrate Fmi homolog Celsr2 can dimerize in an antiparallel arrangement.^5^ This agrees with our work where we show that CAD1–8, which is expressed in cells with a heterologous TM region, binds in *trans* to itself or full-length Fmi in neighboring cells. Nishiguchi et al. also found that the first four cadherin domains of Celsr2 were sufficient to induce bead aggregation,^5^ whereas in our hands, CAD1–4 of Fmi was unable to bind to itself or to full-length Fmi. Furthermore, deletion of any two cadherin domains abrogates binding to full-length Fmi, suggesting that multiple weak *trans* interactions between pairs of cadherin repeats are necessary for robust binding.

Intriguingly, CAD1–8 will bind to full-length Fmi in neighboring cells, but full-length Fmi does not form visible interfaces with Fmi in a neighboring cell unless Fz is also present. We propose a model whereby full-length Fmi exists in a closed conformation that is energetically favored over a conformation allowing homophilic *trans* interactions, resulting in only weak or transient homophilic binding. Binding of Fz to one Fmi molecule opens Fmi making it more energetically favorable to interact in *trans* with a Fmi molecule in the neighboring cell (Figure 7F). Alternatively, rather than Fz binding to Fmi directly causing Fmi to open Fmi, Fz could promote some other change in Fmi behavior such as surface localization or *cis*-clustering,^22^ which might then lead to *trans* interactions being favored. However, neither of these effects are easy to reconcile with the ability of CAD1–8 to mimic Fmi-Fz in promoting interactions in *trans* with full-length Fmi.

Understanding this process in detail will require further study; in particular, we do not yet understand how Fmi and Fz interact. Previous work has shown that deletion of either the N terminus or cytoplasmic domain of Fmi does not affect its ability to co-immunoprecipitate with Fz,^8^ suggesting that the HormR domain, GAIN domain, or TM region of Fmi is important. Other work has implicated the EGF-LamG domains of Fmi in Fz binding,^40^ and our results provide some support for this. There may be multiple interaction interfaces between the two molecules, as we also found that the Fz CRD is necessary but not sufficient to stabilize Fmi:Fmi intercellular complexes.

A second question is why Fz-containing complexes are asymmetric, and why a second Fz molecule is not recruited into the complex in the neighboring cell. We can think of several explanations for this. One is steric hindrance, whereby binding of two Fmi molecules with Fz on one side does not leave sufficient space for a second Fz molecule. However, the CRD of Fz is only 117 amino acids, which is small compared with the extracellular domain of Fmi, so this appears unlikely. An interesting alternative is that Fz in one cell interacts both with Fmi in the same cell and with Fmi in the neighboring cell—possibly via a different domain—and this cannot happen in both directions. Finally, the opening of Fmi in the cell adjacent to Fz may result in a conformational change in Fmi that precludes it from binding to Fz itself.

Furthermore, we envisage that a conformational change in Fmi is the reason why binding of Fz to Fmi in one cell allows Fmi in the neighboring cell to interact with Stbm. Our data show that CAD1–8 in one cell is also sufficient for Fmi in the neighboring cell to recruit Stbm. This is consistent with the long-held view that the ability of complexes to become molecularly asymmetric depends on an asymmetry of Fmi activity.^8,9,21^ Our work implicates the TM region of Fmi in interacting with Stbm. In this context, the identity of Fmi as a member of the aGPCR protein family may be pertinent, as ligand binding to aGPCRs causes conformational changes in the TM domains that allow for the recruitment of substrates.^41,42^ By analogy, we suggest that once Fmi is “activated” by Fmi and Fz in the adjacent cell, intact intracellular loops of Fmi are required for a conformation change in the Fmi TM region that creates a higher affinity Stbm binding site. This site might involve the loops themselves or the TM regions of Fmi.

We previously suggested that the C-terminal cytoplasmic region of Fmi was needed for Stbm recruitment.^21^ However, the new FmiΔIntra construct we have made does not support this conclusion. We speculate that this could be because the new version is tagged with EGFP at the N terminus rather than the C terminus. It is plausible that the presence of EGFP near the TM domains of Fmi in the original truncated construct interferes with the ability of Fmi to interact with Stbm.

Our cell-based system shows that the cytoplasmic core proteins all contribute to complex stability. There are at least two non-exclusive mechanisms via which Stbm and the cytoplasmic proteins stabilize Fmi:Fmi dimers at cell-cell contacts. The first is that their addition to the complexes stabilizes Fmi:Fmi *trans* binding directly, perhaps by inducing a further favorable conformation change. The second is that they promote *cis*-multimerization of dimers into biomolecular condensates (clusters) at cell-cell contacts via mediating multivalent protein-protein interactions, as we have previously suggested.^16^

Interestingly, in our cell system, there is no evidence of stable interactions between proximal and distal complex components in the same cell. Specifically, Dsh is not recruited by Stbm/Pk, and Stbm and Pk are not recruited by Dsh. This stands in contrast to previous work in both cells and *in vitro* that has revealed robust binding between Dsh and Pk and Dsh and Stbm.^31–36^ In the *in vivo* situation, such “mis-localized” proteins could be removed via negative feedback interactions; however, we find no evidence for feedback in our cell system. Instead, we suggest that while Stbm-Dsh and Dsh-Pk are able to bind when not in asymmetric intercellular complexes, once they enter the complex, they undergo conformational changes that block inappropriate interactions, revealing exquisite selectivity of protein-protein interactions in the final assembled complexes.

## Star⋆Methods

### Key Resources Table

**Table:** 

REAGENT or RESOURCE	SOURCE	IDENTIFIER
Antibodies		
Mouse monoclonal anti-Fmi 74	DSHB, Usui et al.^4^	RRID: AB_2619583
Mouse monoclonal anti-Fmi 71	DSHB, Usui et al.^4^	RRID: AB_2619583
Rabbit anti-Fz, affinity purified	Bastock and Strutt^43^	N/A
Rabbit anti-Stbm	Bastock et al.^33^	N/A
Rat anti-Stbm	Strutt and Strutt^21^	N/A
Rat anti-Pk, affinity purified	Strutt et al.^44^	N/A
Rat anti-Dsh	Strutt et al.^45^	N/A
Affinity-purified rat anti-Dgo	Strutt et al.^16^	N/A
Rat anti-Dfz2	Chaudhary et al.^46^	N/A
Rabbit anti-GFP, affinity purified	Abcam	cat#ab6556; RRID: AB_305564
Ratanti-HA 3F10	Roche	cat#1867431; RRID: AB_390918
mouse monoclonal anti-ß-galactosidase 40-1a	DSHB	RRID: AB_2314509
Rabbit anti-Dsh, affinity purified (for western blotting)	Strutt et al.^45^	N/A
Rabbit anti-Stbm (for western blotting)	Rawls and Wolff^47^	N/A
Mouse monoclonal anti-Actin AC40	Sigma-Aldrich	cat#A4700; RRID: AB_476730
Chemicals, peptides, and recombinant proteins		
Alexa Fluor 568 phalloidin	Molecular probes	cat#A-12380
Paraformaldehyde	Agar Scientific	cat#AGR1026
Normal goat serum	Jackson ImmunoResearch	cat#005-000-121; RRID:AB_2336990
Prolong Diamond	Thermo Fisher Scientific	cat#P36965
Schneider’s Drosophila medium	Gibco	cat#21720024
Heat-inactivated fetal bovine serum	Gibco	cat#10082-147
Penicillin-Streptomycin	Sigma-Aldrich	cat#P4333
Effectene transfection reagent	Qiagen	cat#301425
Critical commercial assays		
Pierce West Dura Extended Duration Substrate	Thermo Fisher Scientific	cat#34075
Experimental models: Cell lines		
*D. melanogaster:* Cell line S2	DGRC: 6	FlyBase: FBtc0000006
*D. melanogaster:* Cell line S2R+-NPT005	DGRC: 229	FlyBase: FBtc0000229
*D. melanogaster*: Cell line S2R+-NPT005ΔDshΔStbmΔFz (TKO)	This paper	N/A
Experimental models: Organisms/strains		
*D. melanogaster: fz[P21]*	Adler et al.^48^	BDSC:41787; FlyBase:FBal0004937
*D. melanogaster: stbm[6]*	Wolff and Rubin^49^	BDSC:6918; FlyBase:FBal0062423
*D. melanogaster: pk[pk-sple13]*	Gubb et al.^50^	BDSC:41790; FlyBase:FBal0060943
*D. melanogaster: dsh[1]*	Bloomington Drosophila Stock Center	BDSC:5298; FlyBase:FBal0003138
*D. melanogaster: dgo[380]*	Feiguin et al.^51^	BDSC:41786; FlyBase:FBal0141190
*Ubx-FLP (on X)*	Bloomington Drosophila Stock Center, Emery et al.^52^	BDSC:42718; FlyBase:FBti0150334
*Ubx-FLP (on II)*	Bloomington Drosophila Stock Center^52^	BDSC:42720; FlyBase:FBti0150346
*FRT42 arm-lacZ*	Bloomington Drosophila Stock Center	BDSC:7372; RRID:BDSC_7372
*ubi-mRFP-nls*	Lipsick laboratory (FBrf0210705)	FlyBase:FBti0129786
*FRT80*	Bloomington Drosophila Stock Center, Xu and Rubin^53^	FLYB: FBti0002073
*D. melanogaster: EGFP-fmi* knock-in	Strutt et al.^25^	N/A
*D. melanogaster: pGE-EGFP-fmi*	This paper	N/A
*D. melanogaster: pGE-EGFP-fmiΔIntra* rescue	This paper	N/A
*D. melanogaster: pGE-EGFP-fmiΔIntraΔPBM* rescue	This paper	N/A
*D. melanogaster: pGE-EGFP-fmiΔIL1* rescue	This paper	N/A
*D. melanogaster: pGE-EGFP-fmiΔIL2* rescue	This paper	N/A
*D. melanogaster: pGE-EGFP-fmiΔIL3* rescue	This paper	N/A
Oligonucleotides #1		
CRISPR Targeting Sequence: Dsh #1: CGATGAGACGACGCCGTATCTGG	This paper	N/A
CRISPR targeting sequence: Dsh #2: CCATAACCGACTCGACCATGTCC	This paper	N/A
CRISPR targeting sequence: Stbm #1: AGAATACTACCGCCGTCACGGGG	This paper	N/A
CRISPR targeting sequence: Stbm #2: CCATCTGCACGTTTGCTTACTGG	This paper	N/A
CRISPR targeting sequence: Fz #1: CCCACCCTGATACAGGGGGTCCA	This paper	N/A
CRISPR targeting sequence: Fz #2: CCCGTAAACACCAGACGGGCGTA	This paper	N/A
Recombinant DNA		
pMT-mEGFP-Fmi	This paper	https://doi.org/10.15131/shef.data.24220696.v1
pMT-Fmi-mEGFP	This paper	https://doi.org/10.15131/shef.data.24220696.v1
pMT-Fmi-mApple	This paper	https://doi.org/10.15131/shef.data.24220696.v1
pMT-Fmi-TagBFP2	This paper	https://doi.org/10.15131/shef.data.24220696.v1
pMT-Fmi-3xHA	This paper	https://doi.org/10.15131/shef.data.24220696.v1
pMTΔKpnI-Fmi-EGFP	This paper	https://doi.org/10.15131/shef.data.24220696.v1
pMTΔKpnI-Fmi[ΔCAD2-3]-EGFP	This paper	https://doi.org/10.15131/shef.data.24220696.v1
pMTΔKpnI-Fmi[ΔCAD4-5]-EGFP	This paper	https://doi.org/10.15131/shef.data.24220696.v1
pMTΔKpnI-Fmi[ΔCAD6-7]-EGFP	This paper	https://doi.org/10.15131/shef.data.24220696.v1
pMTΔKpnI-Fmi[ΔCAD8]-EGFP	This paper	https://doi.org/10.15131/shef.data.24220696.v1
pGE-MT-EGFP-Fmi	This paper	https://doi.org/10.15131/shef.data.24220696.v1
pGE-MT-EGFP-Fmi[ΔEGF-LamG]	This paper	https://doi.org/10.15131/shef.data.24220696.v1
pGE-MT-EGFP-Fmi[LamG]	This paper	https://doi.org/10.15131/shef.data.24220696.v1
pGE-MT-EGFP-Fmi[LamG1]	This paper	https://doi.org/10.15131/shef.data.24220696.v1
pGE-MT-EGFP-Fmi[LamG2]	This paper	https://doi.org/10.15131/shef.data.24220696.v1
pGE-MT-EGFP-Fmi[ΔIL1]	This paper	https://doi.org/10.15131/shef.data.24220696.v1
pGE-MT-EGFP-Fmi[ΔIL2]	This paper	https://doi.org/10.15131/shef.data.24220696.v1
pGE-MT-EGFP-Fmi[ΔIL3]	This paper	https://doi.org/10.15131/shef.data.24220696.v1
pMK33ß-CD2Sig-Fmi[CAD1-8]-CD2TM-EGFP	This paper	https://doi.org/10.15131/shef.data.24220696.v1
pMK33ß-CD2Sig-Fmi[CAD1]-CD2TM-EGFP	This paper	https://doi.org/10.15131/shef.data.24220696.v1
pMK33ß-CD2Sig-Fmi[CAD1-2]-CD2TM-EGFP	This paper	https://doi.org/10.15131/shef.data.24220696.v1
pMK33ß-CD2Sig-Fmi[CAD1-4]-CD2TM-EGFP	This paper	https://doi.org/10.15131/shef.data.24220696.v1
pMK33ß-CD2Sig-Fmi[CAD1-8]-EL-CD2TM-EGFP	This paper	https://doi.org/10.15131/shef.data.24220696.v1
pMT-Fz-TagBFP2	This paper	https://doi.org/10.15131/shef.data.24220696.v1
pMT-TagBFP2-Stbm	This paper	https://doi.org/10.15131/shef.data.24220696.v1
pMT-SNAP-Stbm	This paper	https://doi.org/10.15131/shef.data.24220696.v1
pMT-Fz-T2A-Dsh	This paper	https://doi.org/10.15131/shef.data.24220696.v1
pMT-Fz-T2A-Dsh-T2A-Dgo	This paper	https://doi.org/10.15131/shef.data.24220696.v1
pMT-Fz-mEGFP-T2A-Dsh-T2A-Dgo	This paper	https://doi.org/10.15131/shef.data.24220696.v1
pMT-Stbm-T2A-Pk	This paper	https://doi.org/10.15131/shef.data.24220696.v1
pMT-mApple-Stbm-T2A-Pk	This paper	https://doi.org/10.15131/shef.data.24220696.v1
pMT-FzCRD-DFz2-TagBFP2	This paper	https://doi.org/10.15131/shef.data.24220696.v1
pMT-Fz2CRD-Fz-TagBFP2	This paper	https://doi.org/10.15131/shef.data.24220696.v1
pAc-GFP	This paper	https://doi.org/10.15131/shef.data.24220696.v1
Software and algorithms		
ImageJ version 2.0.0-rc-69/1.52p	https://fiji.sc	N/A
GraphPad Prism	www.graphpad.com	N/A
Tissue Analyzer	Aigouy et al.^54^	PMID:20813263
Membrane intensity and Polarity measurement scripts	Strutt et al.^16^	PMID:27926869
QuantifyPolarity version 9	Tan et al.^55^	PMID: 34351416
Image Lab version 4.1	BioRad Laboratories	N/A

### Resource Availability

#### Lead contact

Further information and requests for resources and reagents should be directed to and will be fulfilled by the lead contact, David Strutt (d.strutt@sheffield.ac.uk).

#### Materials availability

Fly strains, cell lines and plasmids reported in this paper will be shared by the lead contact upon request.

### Experimental Model

#### Flies

*Drosophila melanogaster* lines were grown on standard cornmeal/agar/molasses media at 25°C. There are no known differences in the physical and molecular mechanisms of planar polarity in male and female flies, thus flies were not distinguished based on sex. Fly strains are described in FlyBase. *fz*^*P21*^, *stbm*^*6*^, *pk*^*pk-sple13*^, *dsh*^*V26*^ and *dgo*^380^ are null alleles, and *dsh*^1^ gives a strong planar polarity phenotype, but functions normally in Wingless signalling.^56,57^

Full genotypes for figures are:-

Figure 1D: *w; stbm*^*6*^, *Ubx-FLP/stbm*^*6*^; *fz-EGFP FRT80/fz*^*P21*^ *FRT80*

Figure 1E: *w; stbm*^*6*^, *Ubx-FLP/stbm*^*6*^, *Ubx-FLP; P[acman]stbm fz*^*P21*^ *FRT80/fz*^*P21*^ *FRT80*

Figures 1F and 1G red: *w; EGFP-fmi/+*

Figure 1F and 1G green: *w; EGFP-fmi/+; fz*^*P21*^

Figures 1F and 1G orange: *w; stbm*^*6*^ *EGFP-fmi/stbm*^*6*^

Figures 1F and 1G grey: *w; stbm*^*6*^ *EGFP-fmi/stbm*^*6*^; *fz*^*P21*^

Figures 1H and 1I red: *w; EGFP-fmi/+*

Figures 1H and 1I dark green: *Ubx-FLP; EGFP-fmi/+; fz*^*P21*^ *FRT80/ubi-mRFP[nls] FRT80*

Figures 1H and 1I dark orange: *w; stbm*^*6*^ *EGFP-fmi/stbm*^*6*^, *Ubx-FLP; P[acman]stbm FRT80/ubi-mRFP[nls] FRT80*

Figure 1H and 1I grey: *w; stbm*^*6*^ *EGFP-fmi/stbm*^*6*^, *Ubx-FLP; P[acman]stbm fz*^*P21*^ *FRT80/ubi-mRFP[nls] FRT80*

Figures 1H and 1I pale green: *w; stbm*^*6*^ *EGFP-fmi/stbm*^*6*^, *Ubx-FLP; fz*^*P21*^ *FRT80/ubi-mRFP[nls] FRT80*

Figures 1H and 1I pale orange: *w; stbm*^*6*^ *EGFP-fmi/stbm*^*6*^, *Ubx-FLP; P[acman]stbm fz*^*P21*^ *FRT80/ubi-mRFP[nls] fz*^*P21*^ *FRT80*

Figures 3B and S2A red: *w; EGFP-fmi/+*

Figures 3B and S2A pink: *w; EGFP-fmi dgo*^*380*^*/dgo*^*380*^

Figures 3B and S2A blue: *w dsh*^*1*^; *EGFP-fmi/+*

Figures 3B and S2A cyan: *w; pk-sple*^*13*^ *EGFP-fmi/pk-sple*^*13*^

Figures 6B, 6C, S6A, and S6B: *Ubx-FLP; FRT42 pGE-EGFP-fmi/FRT42 arm-lacZ*

Figures 6D, 6E, S6C, and S6D: *Ubx-FLP; FRT42 pGE-EGFP-fmiΔIntra/FRT42 arm-lacZ*

Figures 6F, 6G, S6K, and S6L: *Ubx-FLP; FRT42 pGE-EGFP-fmiΔIL1/FRT42 arm-lacZ*

Figure 6H, 6I, S6N, and S6O: *Ubx-FLP; FRT42 pGE-EGFP-fmiΔIL2/FRT42 arm-lacZ*

Figures 6J, 6K, S6Q, and S6R: *Ubx-FLP; FRT42 pGE-EGFP-fmiΔIL3/FRT42 arm-lacZ*

Figure S6E: *Ubx-FLP; FRT42 pGE-EGFP-fmiΔIntra/FRT42 pGE-EGFP-fmi, arm-lacZ*

Figures S6F–S6I:*Ubx-FLP; FRT42 pGE-EGFP-fmiΔIntraΔPBM/FRT42 arm-lacZ*

Figure S6J: *Ubx-FLP; FRT42 pGE-EGFP-fmiΔIntraΔPBM/FRT42 pGE-EGFP-fmi, arm-lacZ*

Figure S6M: *Ubx-FLP; FRT42 pGE-EGFP-fmiΔIL1/FRT42 pGE-EGFP-fmi, arm-lacZ*

Figure S6P: *Ubx-FLP; FRT42 pGE-EGFP-fmiΔIL2/FRT42 pGE-EGFP-fmi, arm-lacZ*

Figure S6S: *Ubx-FLP; FRT42 pGE-EGFP-fmiΔIL3/FRT42 pGE-EGFP-fmi, arm-lacZ*

#### Cell culture

S2 cells^58^ and S2R+-NPT005 cells,^59^ both of unknown sex, were cultured in Schneider’s *Drosophila* medium, supplemented with 10% heat inactivated fetal bovine serum and 1% Penicillin-Streptomycin at 26°C.

### Method Details

#### Generation of transgenic flies

To generate modified *EGFP-fmi* constructs in a rescue vector, we inserted an *attP* site into the *fmi* locus, replacing an 11 kb region from 26 bp upstream of the start codon to 34 bp downstream of the last common exon, using the targeting vector *pTV[Cherry]*.^60^ Fmi with EGFP downstream of Q355, and full 5’ and 3’ UTRs, all introns, plus 250 bp of upstream intergenic region, was then inserted into the *attP* site in a modified version of the vector *pGE-attB-GMR*.^61^ Internal modifications of the *pGE-fmi* plasmid were made using standard recombineering methods,^62^ replacing specific amino acids with a linker-flanked FRT site (SGGGGSGSSYSLESIGTSSGGGGS). Amino acids deleted are: ΔIL1 2842-2847 (TNSNTL); ΔIL2 2909-2914 (TEMRDI); ΔIL3 2986-2991 (FTLKDH); ΔIntra N3087-L3529; ΔIntraΔPBM: from N3087 to end of ORF. Sequence files are available at https://doi.org/10.15131/shef.data.24220696.v1.

#### Generation of plasmids for tissue culture

Plasmids were made by standard cloning with PCR and restriction enzymes into the pMT-V5/His vector (Clontech) or the pMK33ß vector. For transfection experiments involving Fz or Stbm and the cytoplasmic proteins, T2A self-cleaving peptides were inserted between the open reading frames of each protein within the same plasmid, to ensure that individual cells expressed consistent relative levels of each protein. The T2A peptide sequence (EGRGSLLTCGDVEENPGP) is derived from *Thosea asigna*^63^ and cuts just in front of the final Proline. Sequences for CRISPR were cloned into pL108, which expresses Cas9.^64^ CRISPR sequences were designed with the help of the flyCRISPR (flycrispr.org) or flyRNAi (www.flyrnai.org/crispr) websites and are described in the key resources table.

To make internal modifications of Fmi, a version of the *pGE-fmi* plasmid was made in which the promoter region was replaced by the metallothionein promoter of pMT-V5/His by recombineering, and then further modifications were made, as for the fly versions. Additional deletions were: ΔEGF-Lam (L1421-C2140); ΔLamG (H1576-G1946); ΔLamG1: (H1576-R1736); ΔLamG2: (L1818-G1946).

Sequence files are available at https://doi.org/10.15131/shef.data.24220696.v1.

#### Transfection

Cells were seeded into 24 well plates or 6 well plates at a density of 5 x 10^5^/ml, and grown for 24 hr before transfection with 400 ng or 800 ng DNA using Effectene (Qiagen), according to the manufacturer’s instructions. Cells were grown for 4-6 hr before expression was induced using 350 μM CuSO_4_. Cells were then grown overnight before further processing. Note that for co-transfection experiments with Fmi, we found that all cells that visibly expressed Fmi (expressed from a large plasmid with relatively transfection efficiency) also expressed the co-transfected genes (expressed from smaller plasmids with a higher transfection efficiency).

#### CRISPR deletions in S2R+-NPT005 cells

CRISPR deletions were made as previously described.^65^ Conditioned media was made by splitting confluent cells 1:5 into T75 flasks, and culturing for 3 days, until almost confluent. Media was removed and replaced with 10 ml fresh media. Cells were detached and split 1:2 in new flasks. After a further 16 hr growth, media was collected and filtered through a 0.2 μm filter and stored.

Cells in a 6 well plate were transfected with 180 ng each of two gRNA plasmids and 40 ng of pAc-GFP, using Effectene. After culturing for 4 days, media was removed and replaced with 1 ml PBS containing 1% fetal bovine serum. Cells were detached by pipetting and FACS sorted to select the top 10% of GFP-expressing cells, while excluding the top 1%. 2 cells were placed in each well of 96 well plates, in 250 μl conditioned media. Plates were sealed with Parafilm and cells were cultured for 2-3 weeks, and cells in wells containing single colonies were re-seeded into 96 well plates, and then expanded. Typically 12-18 colonies in each 96 well plate survived.

PCR was used to identify large genomic deletions, and the resulting PCR products were sequenced. Western blotting was then used to confirm a complete loss of protein expression. Deletions identified in the S2R+-NPT005ΔDshB7ΔStbm8ΔFz16 clone are: Dsh: 664 bp deletion of bp 65-728 (between the two gRNAs); Stbm: 452 bp deletion of bp 284-735 (between the two gRNAs) on one chromosome, 5 bp deletion of bp 323-327 on another; Fz: 532 bp deletion of bp 34-565 (between the two gRNAs).

#### Cell aggregation

Cells were seeded and transfected as described above. 16 hr after induction of expression with 350 μM CuSO_4_, cells were detached by vigorous pipetting. Cells were diluted to 8 x 10^5^/ml in media containing 350 μM CuSO_4_, and 250 μl of each cell type (500 μl total) were placed into wells of a non-treated 24 well plate. Cells were allowed to aggregate by swirling at 110 rpm for 90-120 min at 26°C. Cells were then transferred using a 1000 μl pipette with cut-off tip, onto 13 mm coverslips in a fresh 24 well plate, or into CellView cell culture dishes (Greiner) for FRAP. The original wells were washed with 100 μl media containing 350 μM CuSO_4_, and this was added to the coverslips. Cells were allowed to settle for 2 hr before further processing, unless otherwise stated. For FRAP a further 1 ml of media containing 350 μM CuSO_4_ was added to the dishes immediately before imaging.

#### Dissection and immunolabelling of pupal wings

Pupal wings were dissected at 28 hr after puparium formation (APF) at 25°C, or after 27 hr 15 min at 29°C for trichomes. Briefly, pupae were removed from their pupal case and fixed for 35-40 min in 4% paraformaldehyde in PBS, or 55-60 min for Fz immunolabelling. Wings were then dissected and the outer cuticle removed, and were blocked for 1 hr in PBS containing 0.2% Triton X100 (PTX) and 10% normal goat serum. Primary and secondary antibodies were incubated overnight at 4°C in PTX with 10% normal goat serum, and all washes were in PTX. After immunolabelling, wings were post-fixed in 4% paraformaldehyde in PBS for 30 min. Wings were mounted in 25 μl of PBS containing 10% glycerol and 2.5% DABCO, pH7.5.

#### Immunolabelling of cells

Cells on coverslips were washed briefly in PBS, then fixed for 20 min in 4% paraformaldehyde in PBS. They were blocked for 1 hr in PTX and 10% normal goat serum. Primary antibodies were incubated overnight at 4°C, and secondary antibodies for 2-4 hr at RT, in PTX with 10% normal goat serum, and all washes were in PTX. After immunolabelling, wings were post-fixed in 4% paraformaldehyde in PBS for 10 min, and mounted in ProLong Diamond.

For extracellular labelling, cells were washed and blocked after fixation in the absence of detergent, and all antibody labelling was carried out in the absence of detergent. Cells were then permeabilised in PTX and total staining was performed.

#### Imaging of fixed samples

Pupal wings or cells were imaged on a Nikon A1R GaAsP confocal microscope using a 60x NA1.4 apochromatic lens, with a pixel size of 80 nm. Single slices were imaged for cells, and for pupal wings 9 Z-slices separated by 150 nm were imaged and the 3 brightest slices around apicolateral junctions were selected and averaged for each channel in ImageJ.

#### FRAP

For FRAP of pupal wings, a small piece of cuticle was removed from over the pupal wings of 28 hr APF pupae, and the exposed wing was mounted in a drop of Halocarbon 700 oil in a glass-bottomed dish. Images were 256 x 256 pixels, with a pixel size of 100 nm, and a pinhole of 1.2 AU. Up to four elliptical ROIs of 3-4 μm^2^ were selected, either on vertical junctions or on clone boundaries. Three prebleach images were taken at 2 frames/sec, and ROIs were then bleached using a 488 nm Argon laser at 80% with 8 passes (1 sec total time), which resulted in 60-75% bleaching. Immediately following bleaching, 5 images were taken at 5 sec intervals, followed by 10 images at 10 sec intervals, 10 images at 15 sec intervals and 8 images at 30 sec intervals.

For FRAP in cell culture, cells were imaged in CellView dishes, in Schneider’s media. Images were 256 x 256 pixels, with a pixel size of 100 nm, and a pinhole of 1.2 AU. Elliptical ROIs of 3-4 μm^2^ were selected, on cell boundaries where cells expressing EGFP-tagged Fmi formed interfaces with cells expressing Fmi-mApple. Three pre-bleach images were taken at 2 frames/sec, and ROIs were then bleached using a single pass of a 488 nm Argon laser at 5%, which resulted in 60-75% bleaching. Immediately following bleaching, 5 images were taken at 5 sec intervals, followed by 10 images at 10 sec intervals and 26 images at 15 sec intervals. FRAP was carried out between 2-5 hr after plating onto CellView dishes, and there was no correlation between time since plating and fluorescence recovery. Note that we also found no correlation between intensity and recovery with a range of transfected plasmids.

#### Western blotting

Cell lysates were made in RIPA buffer (50 mM Tris-HCl pH7.5, 150 mM NaCl, 1% NP40, 0.5% sodium deoxycholate, 0.1% SDS, 1x protease inhibitors (Roche)), before addition of sample buffer. Western blots were blocked in PBS containing 0.1% Tween-20 and 5 % milk powder, and antibody incubations were in the same buffer. SuperSignal West Dura Extended Duration Substrate (Thermo Scientific) was used for detection and a BioRad ChemiDoc XRS+ was used for imaging.

### Quantification and Statistical Analysis

#### Aggregation experiments

Aggregation experiments were scored blind, by manual counting. 100-200 cells expressing EGFP-tagged Fmi were examined, and the percentage of cells that formed interfaces with cells expressing Fmi-HA was counted. Interfaces were defined as accumulations of proteins visible by eye that extended along the whole contact region, regardless of the length of interface. Blind-scoring was performed on samples from at least three separate sets of aggregation experiments. Percentage recoveries were compared between genotypes using one-way ANOVA. Posthoc tests were used to compare individual samples: Dunnett’s multiple comparison test was used to compare the control to the rest of the genotypes in the experiment; Tukey-Kramer’s multiple comparison test to compare all genotypes within an experiment; and Holm-Šídák’s multiple comparison test was used to compare preselected pairs of samples within an experiment.

#### FRAP analysis

For pupal wings, ImageJ was used to manually reselect and measure bleached regions in each image for each time point. The laser off background was subtracted, and the values were corrected for acquisition bleaching and normalised against the average of the prebleach values. Data were then plotted on an xy graph using Prism (v9 Graphpad), and one-phase exponential curves were fitted to check for goodness of fit. Curves were excluded if the ROI recovery curve failed the "replicates test for lack of fit" in Prism, or if the wing moved out of focus during the course of imaging. Bleached regions within the same wing were averaged. Multiple wings were then combined and two-phase exponential association curves were fitted.

A similar process was carried out for FRAP in cell culture, where ROIs of 1.5-2 μm^2^ were re-selected and measured at each timepoint. We found no evidence for acquisition bleaching during the timecourse of FRAP experiments in cell culture, so no correction was made.

Plateaux and slow and fast half-lives were estimated in Prism, but most of the curves were still rising, so these values were estimates from the extrapolated values and 95% confidence intervals were very wide or could not be calculated. Note that it was not feasible to carry out FRAP for long enough to reach a plateau in most experiments, as our unpublished data suggests two hours would be needed for reliable curve fitting, and there is too much sample movement for this to be a routine procedure. Plateaux in different genotypes tended to converge towards the end of the experiment, but the estimated slow half-lives varied between genotypes. To compare between genotypes, we measured the amount of recovery at a fixed time point, that was equal to the estimated half-life of the slow recovery phase of the wild-type control (210 sec for EGFP-Fmi in pupal wings, or 60 sec for Fmi-EGFP in complexes with Fmi and Fz in cells). This allowed us to quantitively distinguish between genotypes with slow recovery and those with fast recovery.

Recoveries were compared between genotypes using unpaired t-tests, or one-way ANOVA for more than two genotypes. Post hoc tests were used to compare individual samples: Dunnett’s multiple comparison test was used to compare the control to the rest of the genotypes in the experiment; Tukey-Kramer’s multiple comparison test to compare all genotypes within an experiment; and Holm-Šídák’s multiple comparison test was used to compare preselected genotypes within an experiment.

For pupal wings, each experiment was performed on multiple wings from different pupae, which represent biological replicates (n = number of wings). ROIs within a wing were treated as technical replicates and data was averaged per wing. For cell culture each experiment was performed on multiple cells interfaces, and each interface was counted as a biological replicate.

Based on the mean intensity and standard deviation of a control set of wings, we calculated that a sample size of 6 wings per genotype would allow detection of differences of 20% in the means, in a pair-wise comparison, with a power of 0.8 and α 0.05 (using G*Power). As standard deviations were larger for some genotypes, we aimed for 10 wings per genotype.

#### Quantitation of co-localisation along cell interfaces

To quantitate co-localisation along cell interfaces, a 3 pixel-wide line was drawn manually along the interface in the mEGFP channel. ImageJ was used to plot the intensity profile along this line, in the mEGFP and mApple channels. The average intensity in each channel was normalised to 1, and the mEGFP/mApple ratio was determined for each point along the profile. The standard deviation of the ratio was then determined for each interface.

#### Quantitation of extracellular versus total immunolabelling

ImageJ was used to manually draw round cells, using the "total labelling" channel. These ROIs were used to measure mean intensity in the "total labelling" channel and the "extracellular labellingȠ channel. Background values for regions without cells were subtracted, and the channels were ratioed. Ratios of extracellular staining to total staining were normalised to 1 for the control, and the ratios were compared to controls using unpaired t-tests or ANOVA with Dunnett’s multiple comparison test.

#### Quantitation of membrane levels and polarity in pupal wings

Membrane masks were generated in Packing Analyzer,^54^ and MATLAB scripts were used to calculate mean membrane intensity.^16^ Polarity measurements made using QuantifyPolarity.^55^

## Supplementary Material

Supplementary figures

Table S1

Table S2

## Data Availability

All data reported in this paper will be shared by the lead contact upon request. This paper does not report original code. Any additional information required to reanalyse the data reported in this paper is available from the lead contact upon request.

## Highlights

- Frizzled-dependent core planar polarity complexes are intrinsically asymmetric
- Frizzled and Flamingo in one cell bind in *trans* to Flamingo to break molecular symmetry
- Frizzled acts through Flamingo to promote Strabismus recruitment in the adjacent cell
- Strabismus and cytoplasmic core proteins stabilize intercellular polarity complexes

## In brief

Strutt et al. use a cell culture-based system to describe the minimum requirements for molecular symmetry breaking in intercellular core planar polarity complexes. Frizzled in one cell promotes Flamingo homophilic trans interactions. This leads to the recruitment of Strabismus in the neighboring cell, which further stabilizes intercellular complexes.
