# Supplementary material for: Molecular symmetry breaking in the Frizzled-dependent planar polarity pathway
Source: Curr Biol. Author manuscript; Available in PMC 2024 Jun 6. (PMC7616066; doi:10.1016/j.cub.2023.10.071)
Supplement: Supplementary figures [file EMS196569-supplement-Supplementary_figures.pdf]

**Current Biology, Volume *33***

## Supplemental Information

# Molecular symmetry breaking

# in the Frizzled-dependent planar polarity pathway

**Helen Strutt, Samantha Warrington, Amritha Chemmenchery Kokkam Madathil, Tobias Langenhan, and David Strutt**

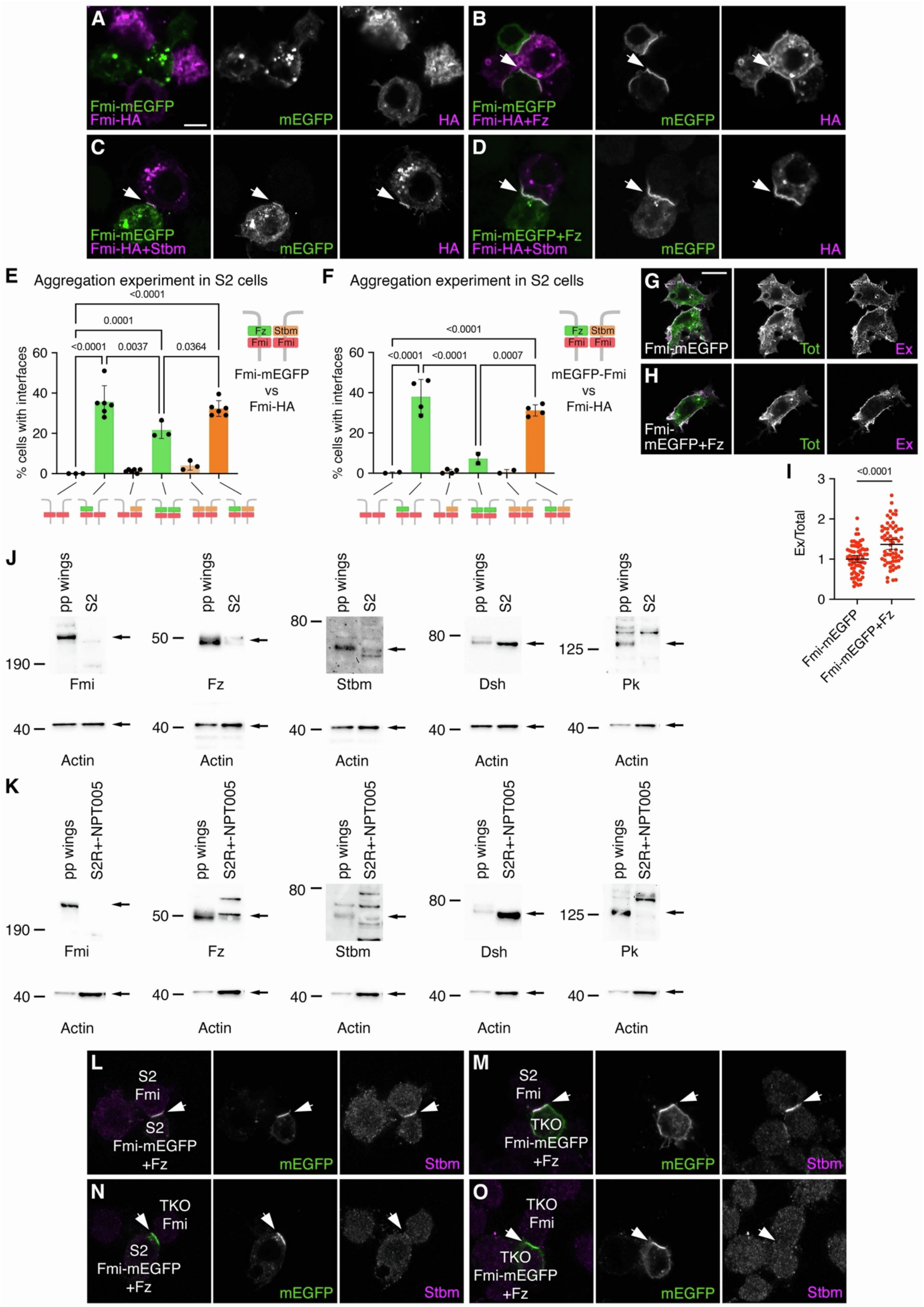

A
Fmi-mEGFP
Fmi-HA
mEGFP
HA
B
Fmi-mEGFP
Fmi-HA+Fz
mEGFP
HA
C
Fmi-mEGFP
Fmi-HA+Stbm
mEGFP
HA
D
Fmi-mEGFP+Fz
Fmi-HA+Stbm
mEGFP
HA
E
Aggregation experiment in S2 cells
% cells with interfaces
<0.0001
0.0001
<0.0001
0.0037
0.0364
Fz
Stbm
Fmi
Fmi
Fmi-mEGFP
vs
Fmi-HA
F
Aggregation experiment in S2 cells
% cells with interfaces
<0.0001
<0.0001
<0.0001
0.0007
mEGFP-Fmi
vs
Fmi-HA
G
Fmi-mEGFP
Tot
Ex
H
Fmi-
mEGFP+Fz
Tot
Ex
I
Ex/Total
<0.0001
Fmi-mEGFP
Fmi-mEGFP+Fz
J
pp wings
S2
190
Fmi
50
Fz
80
Stbm
80
Dsh
125
Pk
40
Actin
K
pp wings
S2R+-NPT005
190
Fmi
50
Fz
80
Stbm
80
Dsh
125
Pk
40
Actin
L
S2
Fmi
S2
Fmi-mEGFP
+Fz
mEGFP
Stbm
M
S2
Fmi
TKO
Fmi-mEGFP
+Fz
mEGFP
Stbm
N
TKO
Fmi
S2
Fmi-mEGFP
+Fz
mEGFP
Stbm
O
TKO
Fmi
TKO
Fmi-mEGFP
+Fz
mEGFP
Stbm

**Figure S1. Asymmetric complex formation in S2 cells. Related to Figure 2.**

(A-D) Aggregation experiments where S2 cells expressing Fmi-mEGFP (A-C) or Fmi-mEGFP and Fz (D) were mixed with cells expressing Fmi-HA (A), Fmi-HA and Fz (B) or Fmi-HA and Stbm (C-D). Cells immunolabelled for GFP (green) or HA (magenta). Arrows point to interfaces between Fmi-mEGFP expressing cells and Fmi-HA expressing cells. Scale bar 5 µm.

(E,F) Quantification of Fmi:Fmi interface formation in S2 cells. Cells expressing Fmi-mEGFP (E) or mEGFP-Fmi (F) were mixed with cells expressing Fmi-HA, in the presence or absence of Fz or Stbm, and mixed as shown in the diagrams below. Graphs shows the mean percentage of one cell population forming visible interfaces with the other (n=3-6), and error bars are sd. Samples were compared using ANOVA with Tukey's multiple comparisons test (selected P values shown).

(G,H) Cells expressing Fmi-EGFP (G) or Fmi-mEGFP and Fz (H), immunolabelled for Fmi in the absence of detergent (‘Ex’, extracellular labelling, magenta) followed by GFP labelling in the presence of detergent (‘Tot’, total labelling, green). Scale bar 10 µm.

(I) Quantitation of the ratio of extracellular labelling to total labelling, error bars are sd. Number of cells scored: Fmi-mEGFP (n=67), Fmi-mEGFP+Fz (n=64). Samples were compared using a Mann-Whitney test.

(J,K) Western blots showing expression of Dsh, Stbm and Fz, but not Fmi and Pk, in S2 cells (G) and S2R+-NPT005 cells (H).

(L-O) S2 cells or S2R+-NPT005 TKO cells expressing Fmi-mEGFP were mixed with cells expressing Fmi, in the presence or absence of Fz in one or other cell type. Immunolabelling for GFP (green) and endogenous Stbm (magenta), reveals recruitment of Stbm to cell-cell interfaces (arrows) of S2 cells expressing Fmi apposed to either S2 cells or TKO cells expressing Fmi and Fz.

See also Tables S1 and S2.

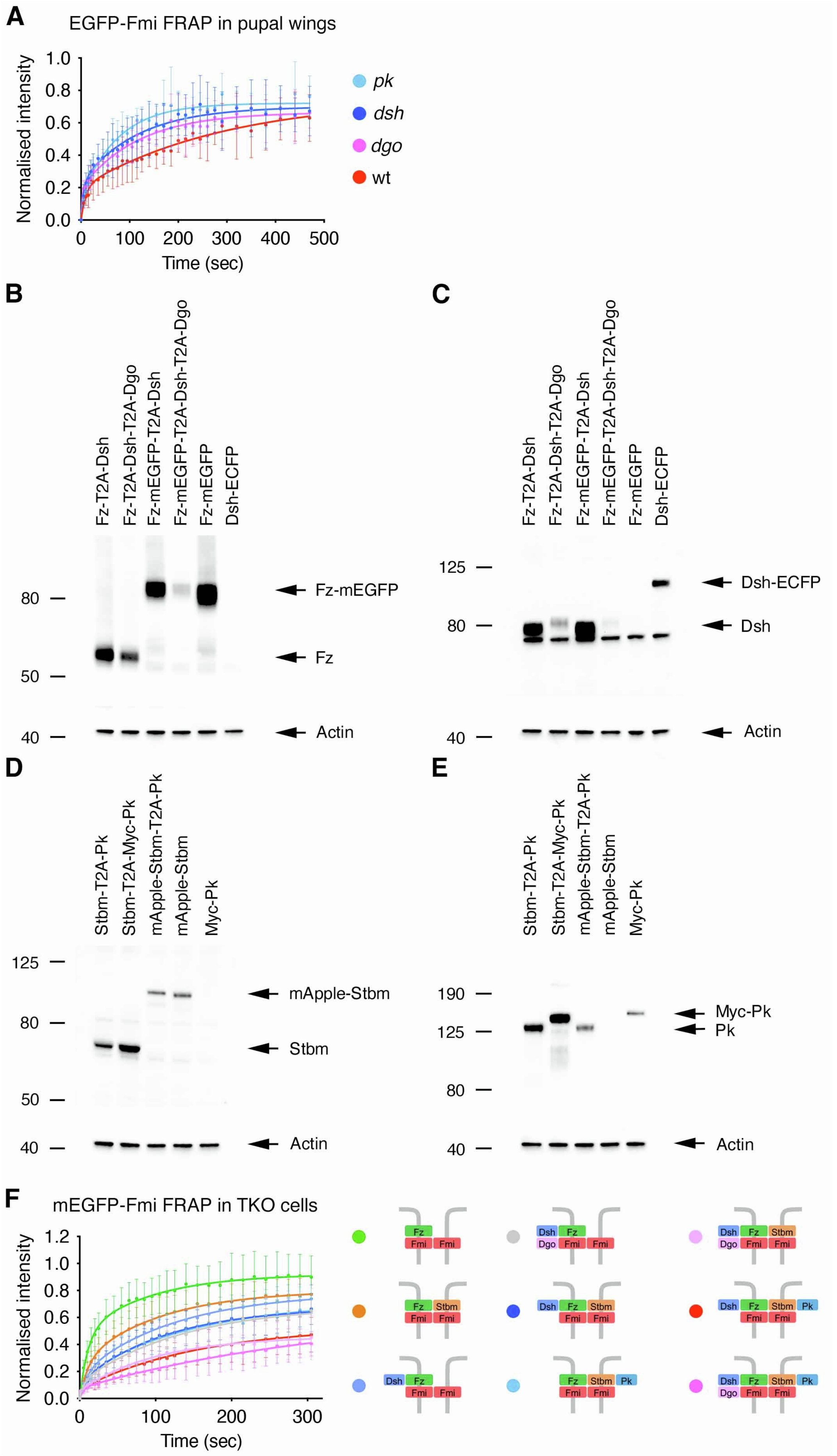

A
EGFP-Fmi FRAP in pupal wings
Normalised intensity
1.0
0.8
0.6
0.4
0.2
0.0
0
100
200
300
400
500
Time (sec)
pk
dsh
dgo
wt
B
Fz-T2A-Dsh
Fz-T2A-Dsh-T2A-Dgo
Fz-mEGFP-T2A-Dsh
Fz-mEGFP-T2A-Dsh-T2A-Dgo
Fz-mEGFP
Dsh-ECFP
80
50
40
Fz-mEGFP
Fz
Actin
C
Fz-T2A-Dsh
Fz-T2A-Dsh-T2A-Dgo
Fz-mEGFP-T2A-Dsh
Fz-mEGFP-T2A-Dsh-T2A-Dgo
Fz-mEGFP
Dsh-ECFP
125
80
40
Dsh-ECFP
Dsh
Actin
D
Stbm-T2A-Pk
Stbm-T2A-Myc-Pk
mApple-Stbm-T2A-Pk
mApple-Stbm
Myc-Pk
125
80
50
40
mApple-Stbm
Stbm
Actin
E
Stbm-T2A-Pk
Stbm-T2A-Myc-Pk
mApple-Stbm-T2A-Pk
mApple-Stbm
Myc-Pk
190
125
80
40
Myc-Pk
Pk
Actin
F
mEGFP-Fmi FRAP in TKO cells
Normalised intensity
1.2
1.0
0.8
0.6
0.4
0.2
0.0
0
100
200
300
Time (sec)
Fz Fmi Fmi
Dsh Fz Dgo Fmi Fmi
Dsh Fz Stbm Dgo Fmi Fmi
Fz Stbm Fmi Fmi
Dsh Fz Stbm Fmi Fmi
Dsh Fz Stbm Pk Fmi Fmi
Dsh Fz Fmi Fmi
Fz Stbm Pk Fmi Fmi
Dsh Fz Stbm Pk Dgo Fmi Fmi

**Figure S2. Expression and stabilising activity of T2A constructs in S2 cells. Related to Figure 3.**

(A) FRAP curves of EGFP-Fmi on cell junctions in 28 hr pupal wings from wild-type (wt) (red), *dsh* (dark blue), *dgo* (pink) and *pk* (pale blue) mutants. Two-phase exponential curves were fitted, error bars are sd.

(B-E) Western blots showing cleavage of Fz-T2A constructs and Stbm-T2A constructs. Extracts from S2 cells transfected with the constructs indicated, westerns probed with Fz antibody (B), Dsh antibody (C), Stbm antibody (D) or Pk antibody (E). Western blots were also probed with Actin antibody as loading control. All constructs are cleaved as expected at the T2A site, but note that expression from the Fz-T2A-Dsh-T2A-Dgo constructs is significantly reduced.

(F) FRAP curves of mEGFP-Fmi in S2R+-NPT005 TKO cells, on interfaces with Fmi-mApple expressing cells, with Stbm, Dsh, Pk and/or Dgo co-transfected as indicated in key to the right. Two-phase exponential curves were fitted, error bars are sd.

See also Table S2.

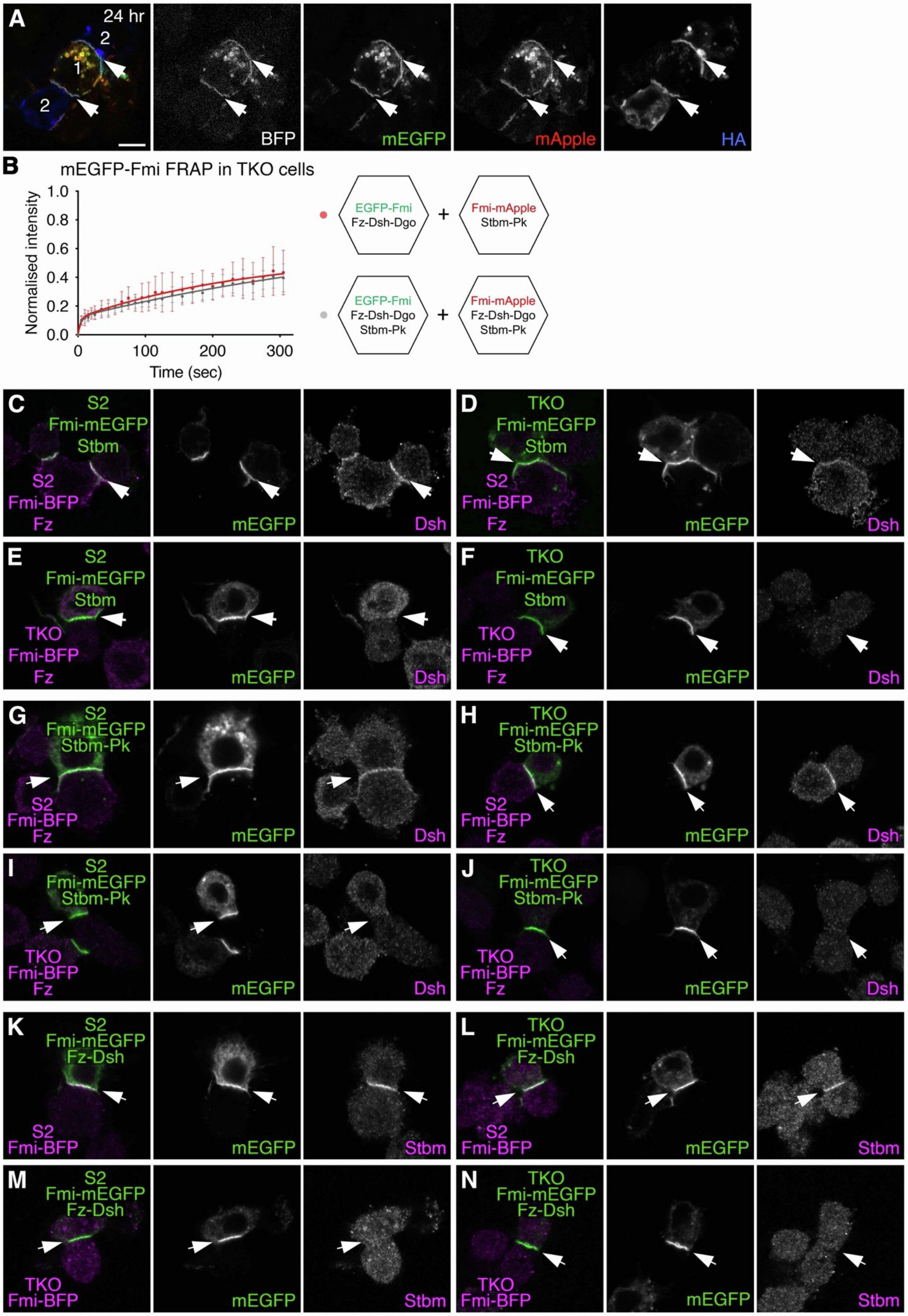

A
24 hr
1
2
2
BFP
mEGFP
mApple
HA
B
mEGFP-Fmi FRAP in TKO cells
Normalised intensity
1.0
0.8
0.6
0.4
0.2
0.0
0
100
200
300
Time (sec)
EGFP-Fmi
Fz-Dsh-Dgo
+
Fmi-mApple
Stbm-Pk
EGFP-Fmi
Fz-Dsh-Dgo
Stbm-Pk
+
Fmi-mApple
Fz-Dsh-Dgo
Stbm-Pk
C
S2
Fmi-mEGFP
Stbm
S2
Fmi-BFP
Fz
mEGFP
Dsh
D
TKO
Fmi-mEGFP
Stbm
S2
Fmi-BFP
Fz
mEGFP
Dsh
E
S2
Fmi-mEGFP
Stbm
TKO
Fmi-BFP
Fz
mEGFP
Dsh
F
TKO
Fmi-mEGFP
Stbm
TKO
Fmi-BFP
Fz
mEGFP
Dsh
G
S2
Fmi-mEGFP
Stbm-Pk
S2
Fmi-BFP
Fz
mEGFP
Dsh
H
TKO
Fmi-mEGFP
Stbm-Pk
S2
Fmi-BFP
Fz
mEGFP
Dsh
I
S2
Fmi-mEGFP
Stbm-Pk
TKO
Fmi-BFP
Fz
mEGFP
Dsh
J
TKO
Fmi-mEGFP
Stbm-Pk
TKO
Fmi-BFP
Fz
mEGFP
Dsh
K
S2
Fmi-mEGFP
Fz-Dsh
S2
Fmi-BFP
mEGFP
Stbm
L
TKO
Fmi-mEGFP
Fz-Dsh
S2
Fmi-BFP
mEGFP
Stbm
M
S2
Fmi-mEGFP
Fz-Dsh
TKO
Fmi-BFP
mEGFP
Stbm
N
TKO
Fmi-mEGFP
Fz-Dsh
TKO
Fmi-BFP
mEGFP
Stbm

**Figure S3. Analysis of complex sorting in aggregated cells. Related to Figure 4.**

(A) S2R+-NPT005 TKO cells expressing Fmi-TagBFP, Fz-mEGFP-Dsh-Dgo and mApple-Stbm-Pk mixed with cells expressing Fmi-HA, Fz-Dsh-Dgo and Stbm-Pk, and allowed to interact for 24 hr. Cells immunolabelled for HA (blue), and showing BFP (not in overlay), mEGFP (green) and mApple (red) fluorescence. Arrows point to typical interfaces between the two cell types, showing co-localisation along the entire interface. Scale bar 5 μm.

(B) FRAP curves of mEGFP-Fmi in S2R+-NPT005 TKO cells, on interfaces with Fmi-mApple expressing cells. Red bar shows interfaces between cells expressing EGFP-Fmi, Fz, Dsh and Dgo mixed with cells expressing Fmi-mApple,Stbm and Pk, grey bar shows interfaces between cells expressing EGFP-Fmi and cells expressing mApple-Fmi, where both cell types co-express Fz, Dsh, Dgo, Stbm and Pk. Two-phase exponential curves were fitted, error bars are sd.

(C-F) S2 cells or S2R+-NPT005 TKO cells expressing Fmi-mEGFP and Stbm were mixed with cells expressing Fmi-TagBFP and Fz. Immunolabelling for GFP (green) and Dsh (magenta) reveals recruitment of endogenous Dsh to the interfaces (arrows) of S2 cells expressing Fmi-Fz apposed to either S2 cells or TKO cells expressing Fmi-Stbm (C,D), showing that Dsh is recruited into distal complexes. No recruitment of endogenous Dsh in S2 cells to cell interfaces is seen (arrows) if TKO cells expressing Fmi-Fz were mixed with S2 or TKO cells expressing Fmi-Stbm (E,F).

(G-J) S2 cells or S2R+-NPT005 TKO cells expressing Fmi-mEGFP and Stbm-Pk were mixed with cells expressing Fmi-TagBFP and Fz. Immunolabelling for GFP (green) and Dsh (magenta) reveals recruitment of endogenous Dsh to cell-cell interfaces (arrows) of S2 cells expressing Fmi-Fz apposed to either S2 cells or TKO cells expressing Fmi-Stbm-Pk (G,H), showing that Dsh is recruited into distal complexes. No recruitment of endogenous Dsh in S2 cells to cell interfaces is seen (arrows) if TKO cells expressing Fmi-Fz are apposed to S2 or TKO cells expressing Fmi-Stbm-Pk (I,J).

(K-N) S2 cells or S2R+-NPT005 TKO cells expressing Fmi-mEGFP and Fz-Dsh were mixed with cells expressing Fmi-TagBFP. Immunolabelling for GFP (green) and Stbm (magenta) reveals recruitment of endogenous Stbm to cell-cell interfaces (arrows) of S2 cells expressing Fmi apposed to either S2 cells or TKO cells expressing Fmi-Fz-Dsh (K,L) , showing that Stbm is recruited on the opposite side to Fmi-Fz-Dsh. No recruitment of endogenous Stbm in S2 cells to interfaces is seen (arrows) if S2 or TKO cells expressing Fmi-Fz-Dsh are apposed to TKO cells expressing Fmi (M,N).

See also Table S2.

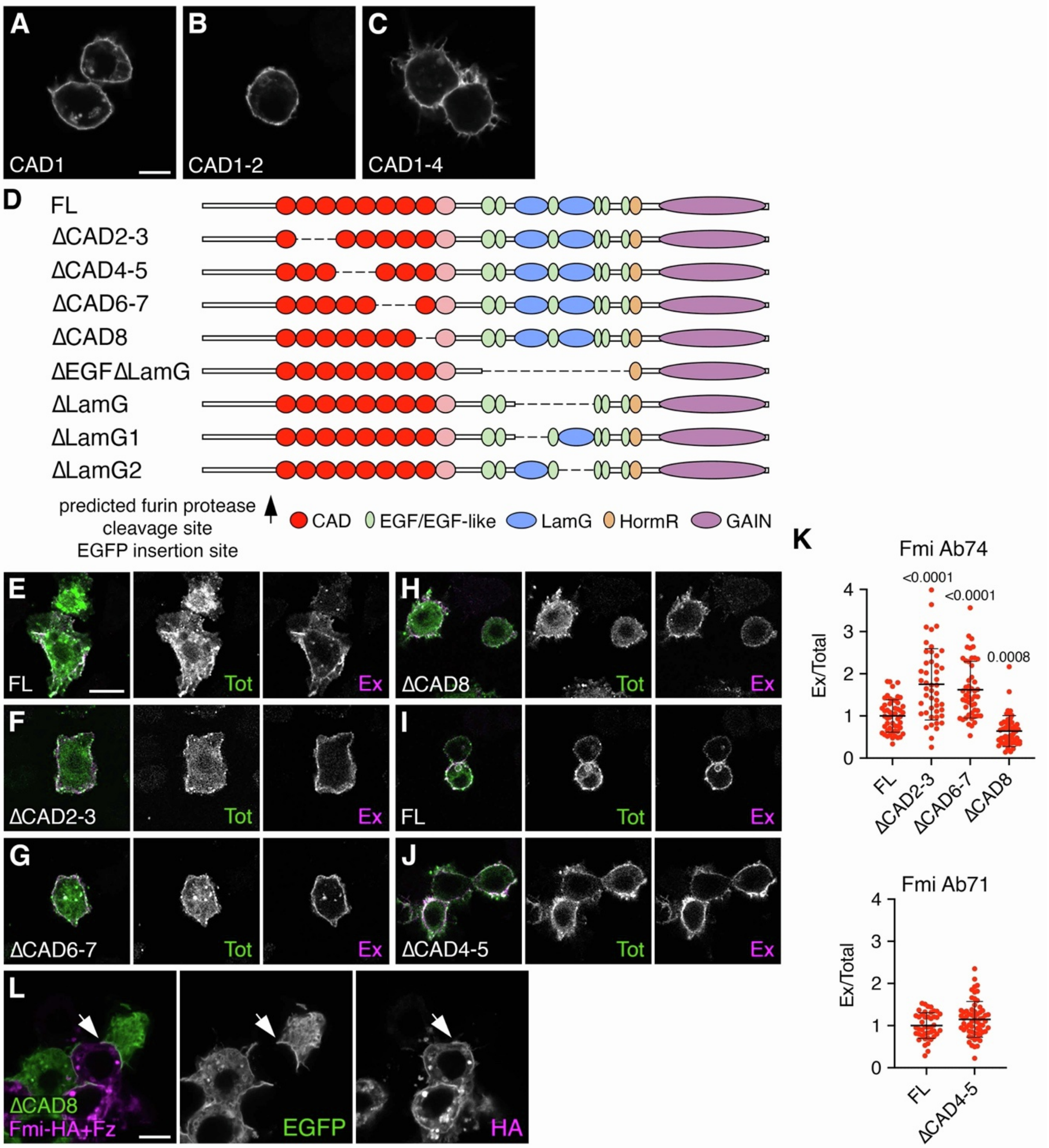


**Figure S4. Dissection of Fmi homophilic binding in the presence of Fz. Related to Figure 5.**

(A-C) S2R+-NPT005 TKO cells expressing CD2Sig-Fmi[CAD1]CD2TM-EGFP (A), CD2Sig-Fmi[CAD1-2]-CD2TM-EGFP (B) or CD2Sig-Fmi[CAD1-4]-CD2TM-EGFP (C), immunolabelled for GFP. Scale bar 5 μm.

(D) Schematic of the extracellular domain of Fmi, and positions of deletions of the cadherin domains and EGF-LamG domains.

(E-J) S2 cells expressing Fmi-EGFP with cadherin deletions as indicated. Cells immunolabelled for Fmi in the absence of detergent ('Ex', extracellular labelling, magenta) followed by GFP labelling in the presence of detergent ('Tot', total labelling, green). (E-H) Labelling with Fmi monoclonal antibody 74, that recognises an epitope in the extracellular domain, note that Fmi[∆CAD4-5] is not recognised by this antibody. (I,J) Labelling with Fmi monoclonal antibody 71, that also recognises an epitope in the extracellular domain, note that Fmi[∆CAD2-3] is not recognised by this antibody. Scale bar 10 μm.

(K) Quantitation of the ratio of extracellular labelling to total labelling for the Fmi cadherin deletions, error bars are sd. Number of cells scored for top graph: full-length (FL) (n=52), ∆CAD2-3 (n=47), ∆CAD6-7 (n=49), ∆CAD8 (n=50); for bottom graph: FL (n=44), ∆CAD4-5 (n=61). Samples were compared to FL Fmi using ANOVA with Kruskal-Wallis multiple comparisons test, or using a Mann-Whitney test.

(L) S2 cells expressing Fmi[∆CAD8]-EGFP, mixed with cells expressing Fmi-HA and Fz. Cells immunolabelled for GFP (green) or HA (magenta). Arrows point to weak interfaces between the two cell types. Scale bar 5 μm.

See also Tables S1 and S2.

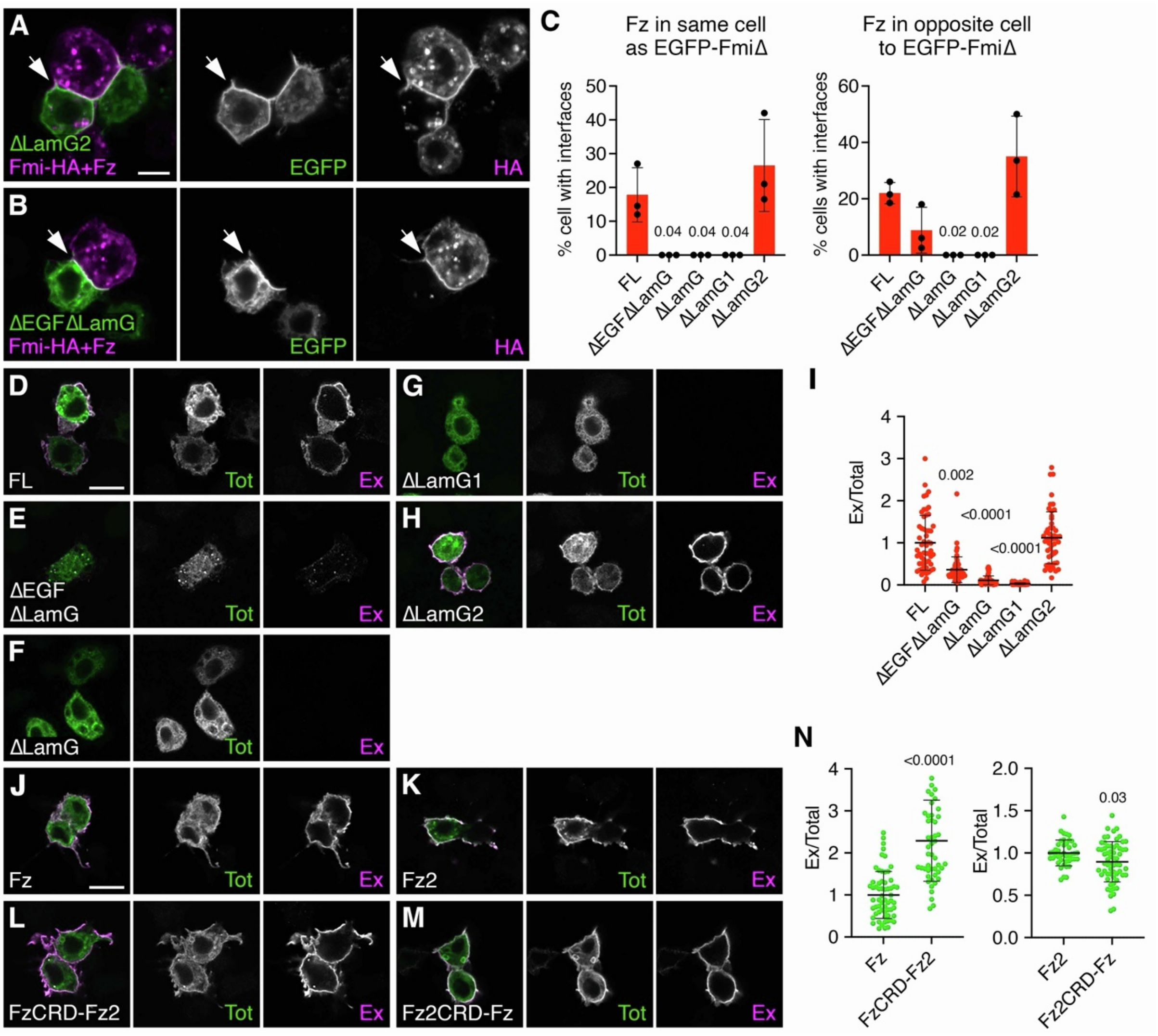


**Figure S5. Dissection of Fmi interactions with Fz. Related to Figure 5.**

(A,B) S2R+-NPT005 TKO cells expressing EGFP-Fmi[ΔLamG2] (A) or EGFP-Fmi[ΔEGFΔLamG] (B), mixed with cells expressing Fmi-HA and Fz. Cells immunolabelled for GFP (green) or HA (magenta). Arrows point to interfaces between the two cell types. Scale bar 5 µm.

(C) Graph showing the mean percentage of cells expressing EGFP-Fmi with EGF-LamG deletions forming visible interfaces with expressing Fmi-HA, in the presence of absence of Fz in one or other cell (n=3). Error bars are sd. Samples were compared to FL Fmi using ANOVA with Dunnett's multiple comparisons test.

(D-H) S2 cells expressing EGFP-Fmi with EGF-LamG deletions as indicated. Cells immunolabelled for GFP in the absence of detergent (‘Ex’, extracellular labelling, magenta)

followed by Fmi labelling in the presence of detergent (‘Tot’, total labelling, green). Scale bar 10 μm.

(I) Quantitation of the ratio of extracellular labelling to total labelling for the Fmi EGF-LamG deletions, error bars are sd. Number of cells scored: FL (n=55), ΔEGFΔLamG (n=58), ΔLamG (n=70), ΔLamG1 (n=104), ΔLamG2 (n=51). Samples were compared to FL Fmi using ANOVA with Kruskal-Wallis multiple comparisons test.

(J-M) S2R+-NPT005 TKO cells expressing Fz chimaeras as indicated. Cells immunolabelled for Fz (J,L) or DFz2 (K,M) in the absence of detergent (extracellular labelling, magenta, note that both antibodies recognise extracellular epitopes) followed by Fz or DFz2 labelling in the presence of detergent (total labelling, green). Scale bar 10 μm.

(N) Quantitation of the ratio of extracellular labelling to total labelling for the Fz/DFz2 chimaeras, error bars are sd. Number of cells scored: Fz (n=61), FzCRD-Fz2 (n=51), Fz2 (n=43), Fz2CRD-Fz (n=62). Samples were compared to FL Fmi using a Mann-Whitney test.

See also Tables S1 and S2.

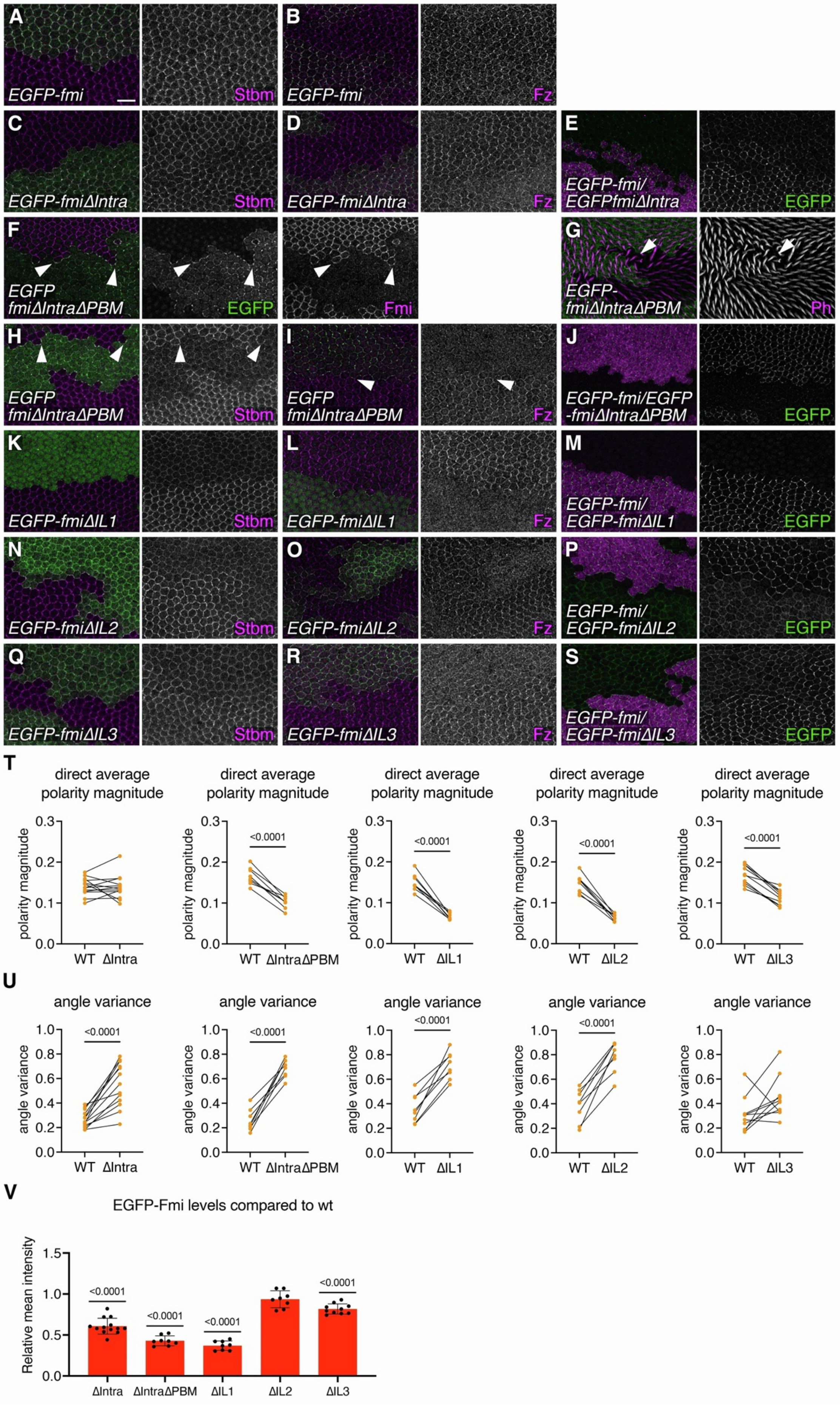
A
EGFP-fmi
Stbm
B
EGFP-fmi
Fz
C
EGFP-fmiΔIntra
Stbm
D
EGFP-fmiΔIntra
Fz
E
EGFP-fmi/
EGFPfmiΔIntra
EGFP
F
EGFP
fmiΔIntraΔPBM
EGFP
Fmi
G
EGFP-
fmiΔIntraΔPBM
Ph
H
EGFP
fmiΔIntraΔPBM
Stbm
I
EGFP
fmiΔIntraΔPBM
Fz
J
EGFP-fmi/EGFP
-fmiΔIntraΔPBM
EGFP
K
EGFP-fmiΔIL1
Stbm
L
EGFP-fmiΔIL1
Fz
M
EGFP-fmi/
EGFP-fmiΔIL1
EGFP
N
EGFP-fmiΔIL2
Stbm
O
EGFP-fmiΔIL2
Fz
P
EGFP-fmi/
EGFP-fmiΔIL2
EGFP
Q
EGFP-fmiΔIL3
Stbm
R
EGFP-fmiΔIL3
Fz
S
EGFP-fmi/
EGFP-fmiΔIL3
EGFP
T
direct average polarity magnitude
polarity magnitude
0.0
0.1
0.2
0.3
<0.0001
WT
ΔIntra
ΔIntraΔPBM
ΔIL1
ΔIL2
ΔIL3
U
angle variance
0.0
0.2
0.4
0.6
0.8
1.0
<0.0001
V
EGFP-Fmi levels compared to wt
Relative mean intensity
0.0
0.5
1.0
1.5
<0.0001
ΔIntra
ΔIntraΔPBM
ΔIL1
ΔIL2
ΔIL3

**Figure S6. Characterisation of EGFP-Fmi variants in pupal wings. Related to Figure 6.**

(A-S) Pupal wings carrying clones of *EGFP-fmi* variants, juxtaposed to wild-type tissue (A-D,F-I,K,L,N,O,Q,R), or juxtaposed to wild-type *EGFP-fmi* (E,J,M,P,S). (A-F,H-S) aged 28h at 25°C, (G) aged 29°C for 27 hr 15 min. (A,B) wild-type *EGFP-fmi*, (C-E) *EGFP-fmiΔIntra*, (F-J) *EGFP-fmiΔIntraΔPBM*, (K-M) *EGFP-fmiΔIL1*, (N-P) *EGFP-fmiΔIL2*, (Q-S) *EGFP-fmiΔIL3*. (A,C,H,K,N,Q) Clones immunolabelled for GFP (green) and Stbm (magenta). (B,D,I,L,O,R) Clones immunolabelled for Fz (magenta) and showing EGFP fluorescence (green). (E,J,M,P,S) Clones immunolabelled for ß-galactosidase (red) and showing EGFP fluorescence (green). (F) Clones immunolabelled for Fmi (red) and showing EGFP fluorescence (green). (G) Clones immunolabelled for GFP (green) and labelled for Phalloidin (magenta). Arrowheads point to accumulation of EGFP-Fmi on clone boundaries. Arrows point to non-autonomous trichome swirling, on the distal side of *fmiΔIntraΔPBM* clones. Scale bar 10 µm.

(T-V) Quantitation of polarity and membrane levels in clones of *EGFP-fmi* variants compared to *EGFP-fmi* twinclones in the same wing. (T) Direct average polarity magnitude; (U) angle variance, based on Stbm immunolabelling. (V) Relative membrane levels of EGFP, error bars are sd. Number of wings = 13 (*fmiΔIntra*), 8 (*fmiΔIntraΔPBM*), 8 (*fmiΔIL1*), 8 (*fmiΔIL2*), 10 (*fmiΔIL3*). Values in mutant clone tissue compared to wild type tissue in the same wing using a paired t-test (T,U ) or a one-sample t and Wilcoxon test (V).

See also Table S1.

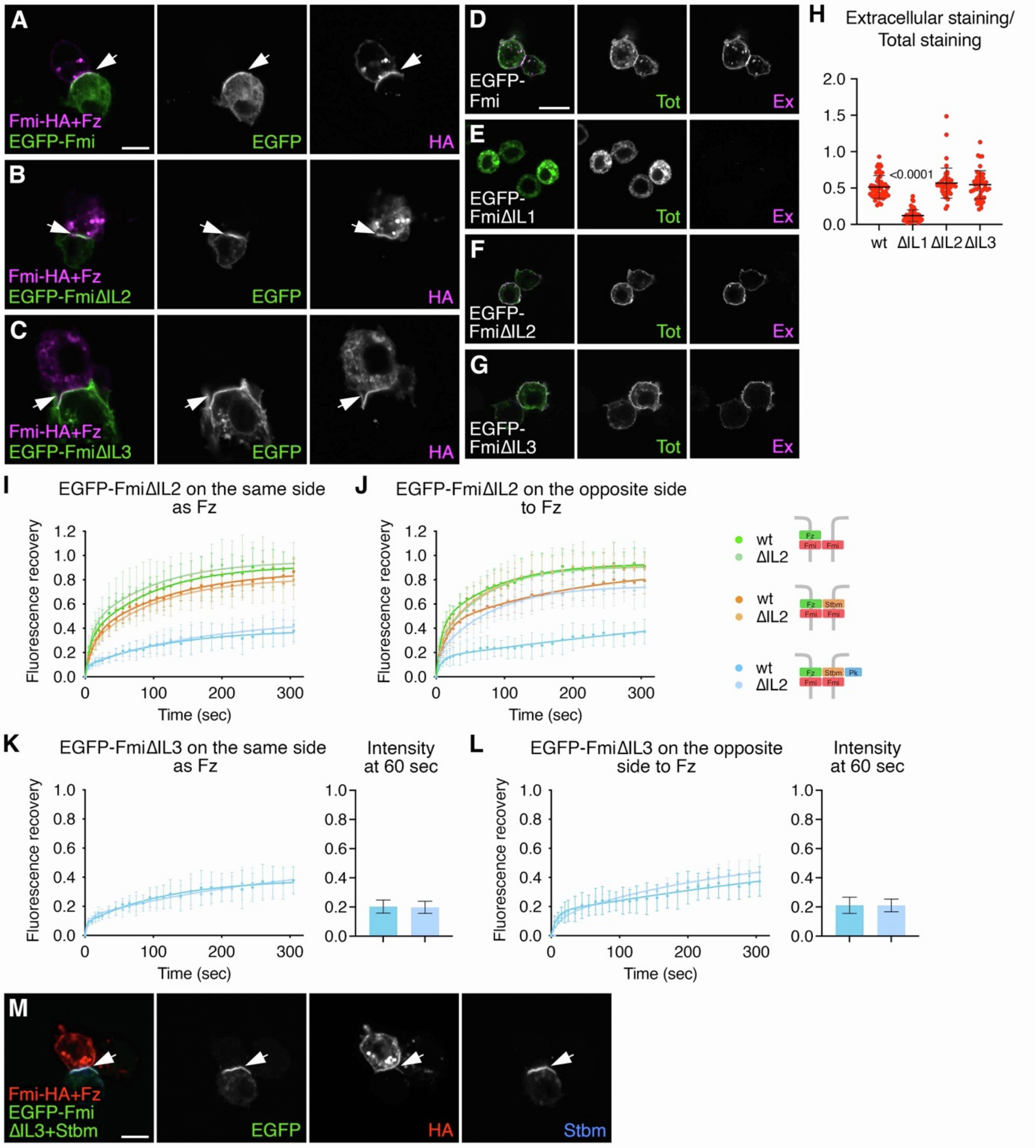


**Figure S7. Dissecting the role of the intracellular loops of Fmi. Related to Figure 7.**

(A-C) S2R+-NPT005 TKO cells expressing EGFP-Fmi (A), EGFP-Fmi[ΔIL2] (B) or EGFP-Fmi[ΔIL3] (C) were mixed with cells expressing Fmi-HA and Fz. Cells immunolabelled for GFP (green) and HA (magenta). Arrows point to interfaces between EGFP-Fmi expressing cells and Fmi-HA expressing cells. Scale bar 5 µm.

(D-G) S2 cells expressing EGFP-Fmi with intracellular loop mutations as indicated. Cells immunolabelled for Fmi in the absence of detergent (‘Ex’, extracellular labelling, magenta)

followed by GFP labelling in the presence of detergent (‘Tot’, total labelling, green). Scale bar 10 µm.

(H) Quantitation of the ratio of extracellular labelling to total labelling for the Fmi intracellular loop mutations, error bars are sd. Number of cells scored: FL (n=53), ΔIL1 (n=52), ΔIL2 (n=49), ΔIL3 (n=48). Samples were compared to wild type Fmi using ANOVA with Kruskal-Wallis multiple comparisons test.

(I,J) FRAP curves of EGFP-Fmi (darker lines) and EGFP-FmiΔIL2 (paler lines) in S2R+-NPT005 TKO cells, on interfaces with Fmi-mApple expressing cells. Green lines show FRAP on interfaces between cells expressing Fmi and cells expressing Fz-Fmi, where the Fz is on the same side (I) or the opposite side (J) to EGFP-Fmi or EGFP-FmiΔIL2. Orange lines show FRAP on Fz-Fmi:Fmi-Stbm interfaces, and blue lines show FRAP on Fz-Fmi:Fmi-Stbm-Pk interfaces. Two-phase exponential curves were fitted, error bars are sd.

(K,L) FRAP curves of EGFP-Fmi (darker blue) and EGFP-FmiΔIL3 (paler blue) in S2R+-NPT005 TKO cells (left) or fluorescence recovery 60 sec after bleaching (right), on interfaces with Fmi-mApple expressing cells. One cell expresses Fmi-Stbm-Pk, and the other expresses Fz-Fmi, where the Fz is on the same side (K) or the opposite side (L) to EGFP-Fmi or EGFP-FmiΔIL3. (K) n=11 (dark blue), n=10 (pale blue). (L) n=9 (dark blue), n=11 (pale blue). Two-phase exponential curves were fitted, error bars are sd. Samples were compared using an unpaired t-test, no significant differences were seen.

(M) S2R+-NPT005 TKO cells expressing EGFP-FmiΔIL3 and Stbm were mixed with cells expressing Fmi-HA and Fz. Cells immunolabelled for HA (red) or Stbm (blue) and showing EGFP fluorescence (green). Arrows point to interfaces between EGFP-Fmi expressing cells and Fmi-HA expressing cells. Scale bar 5 µm.

See also Tables S1 and S2.
